# A BpbZIP4 transcription factor enhances drought resistance and root development in *Betula platyphylla*: insights into a gene regulatory network

**DOI:** 10.1093/hr/uhag002

**Published:** 2026-01-07

**Authors:** Hu Sun, Kaixing Pang, Xuemei Zhou, Luyao Wang, Binrong Li, Jiaxue Wei, Huiyan Guo, Yucheng Wang

**Affiliations:** College of Forestry, Shenyang Agricultural University, Shenyang, Liaoning 110866, China; The Key Laboratory of Forest Tree Genetics, Breeding and Cultivation of Liaoning Province, Shenyang Agricultural University, Shenyang, Liaoning 110866, China; College of Forestry, Shenyang Agricultural University, Shenyang, Liaoning 110866, China; The Key Laboratory of Forest Tree Genetics, Breeding and Cultivation of Liaoning Province, Shenyang Agricultural University, Shenyang, Liaoning 110866, China; College of Forestry, Shenyang Agricultural University, Shenyang, Liaoning 110866, China; The Key Laboratory of Forest Tree Genetics, Breeding and Cultivation of Liaoning Province, Shenyang Agricultural University, Shenyang, Liaoning 110866, China; College of Forestry, Shenyang Agricultural University, Shenyang, Liaoning 110866, China; The Key Laboratory of Forest Tree Genetics, Breeding and Cultivation of Liaoning Province, Shenyang Agricultural University, Shenyang, Liaoning 110866, China; College of Forestry, Shenyang Agricultural University, Shenyang, Liaoning 110866, China; The Key Laboratory of Forest Tree Genetics, Breeding and Cultivation of Liaoning Province, Shenyang Agricultural University, Shenyang, Liaoning 110866, China; College of Forestry, Shenyang Agricultural University, Shenyang, Liaoning 110866, China; The Key Laboratory of Forest Tree Genetics, Breeding and Cultivation of Liaoning Province, Shenyang Agricultural University, Shenyang, Liaoning 110866, China; College of Forestry, Shenyang Agricultural University, Shenyang, Liaoning 110866, China; The Key Laboratory of Forest Tree Genetics, Breeding and Cultivation of Liaoning Province, Shenyang Agricultural University, Shenyang, Liaoning 110866, China; College of Forestry, Shenyang Agricultural University, Shenyang, Liaoning 110866, China; The Key Laboratory of Forest Tree Genetics, Breeding and Cultivation of Liaoning Province, Shenyang Agricultural University, Shenyang, Liaoning 110866, China

## Abstract

Drought is a major abiotic stress that poses a significant threat to plants. Basic leucine zipper (bZIP) transcription factors (TFs) are important for plant stress signal transduction. However, the specific functions and molecular mechanisms of bZIP TFs under drought stress are still unclear. In this study, a BpbZIP4 TF of *Betula platyphylla* (birch) that responds strongly to drought stress was identified. Transgenic birch plants with *BpbZIP4* overexpression and RNA interference were developed for gain- and loss-of-function assays. Results from phenotypic, staining, and physiological analyses showed that *BpbZIP4* significantly enhances drought resistance and promotes root growth in birch. A four-layer drought-responsive gene regulatory network (GRN) was constructed based on *BpbZIP4* transgenic lines. Chromatin immunoprecipitation-polymerase chain reaction (ChIP-PCR) and quantitative reverse transcription-polymerase chain reaction (qRT-PCR) assays verified the putative interactions among genes at different hierarchical levels, confirming the reliability of the GRN. TF-Centered Y1H, ChIP, and β-glucuronidase (GUS) assays revealed that BpbZIP4 regulates the expression of second-layer TFs in the GRN by binding to two novel elements and one photosynthesis-responsive element. Furthermore, six randomly selected second-layer GRN TFs (BpMYB61, BpBEL1, BpWOX4, BpbHLH100, BpZAT11, and BpHB17), when transformed into birch plants, significantly influence birch’s drought tolerance. These results indicate that BpbZIP4 regulates second-layer TFs, thereby hierarchically relaying signals to bottom-layer functional genes, engaging multiple biological pathways, and ultimately enhancing drought resistance in birch. Collectively, these findings clarify the drought regulatory mechanism of BpbZIP4 and identify additional key genes for breeding drought-resistant birch varieties.

## Introduction

Drought, as a common type of abiotic stress affecting ecosystems, leads to water deficiency, nutritional imbalances, ion toxicity, and oxidative stress in plants [1, 2]. These adverse effects significantly hinder the growth, reproduction, and adaptability of tree species [3]. Plants have developed diverse physiological and molecular strategies to cope with the adverse effects of drought [4]. Transcription factors (TFs) serve as molecular switches that govern the transcription of genes associated with plant stress responses, and they play a pivotal role in supporting plant adaptation to various developmental stages and environmental challenges [5].

Among plant TF families, the basic leucine zipper (bZIP) stands out as one of the largest, and it fulfills a critical role in governing plant growth and development, encompassing events such as seed germination, root elongation, and flower formation [6–10]. Some research has demonstrated that bZIP TFs are pivotal to plants’ adaptation to abiotic stress, particularly regarding drought conditions [11]. For example, overexpressing *ZmbZIP89* in transgenic maize lines resulted in significantly higher photosynthetic rates, stomatal conductance, and transpiration in comparison with those of control plants under drought conditions. Furthermore, *ZmbZIP89* activates the expression of *ZmPRX47* to regulate reactive oxygen species (ROS) homeostasis, which contributes to increased lateral root length and enhanced drought resistance [12]. A drought-responsive bZIP TF, SibZIP67, from *Foxtail millet* was identified, and its ectopic overexpression (OE) lines in *Arabidopsis* were generated. The results showed that SibZIP67 enhances the ability of plants to withstand drought through increasing both antioxidant enzyme activity and the expression of genes linked to drought tolerance [13]. Another study showed that the expression of *CaADBZ1* was specifically activated by dehydration and treatment with exogenous abscisic acid (ABA). Further analysis demonstrated that *CaADBZ1* decreases water transpiration rates and ultimately improves drought resistance in transgenic pepper and *Arabidopsis* plants by positively influencing ABA sensitivity and the expression of dehydration-responsive genes [14]. HvbZIP21 from barley has been identified as a factor that enhances drought resistance in plants by regulating stomatal opening and boosting the scavenging capacity for ROS when overexpressed in *Arabidopsis* [15]. The OE of *GmTRAB1* in soybean enhanced the drought resistance of transgenic *Arabidopsis* and soybean by increasing proline content, boosting the activity of antioxidant enzymes, and reducing the deposition of ROS and malondialdehyde (MDA) [16]. The above studies found that bZIP TFs play an important role in the drought resistance of plants; however, their regulatory mechanisms have not yet been fully elucidated.

The drought regions in China cover ~3.5 million square kilometers, which accounts for ~36% of the nation’s total land area [17,18]. Therefore, it is indispensable to develop new varieties of forest trees that are tolerant to drought conditions for sustainable forestry production. As a pioneer tree species, *Betula platyphylla* (birch) holds significant ecological value. Furthermore, it is characterized by white bark and straight, upright trunks—attributes that contribute to its considerable economical and ornamental benefits [19]. Here, a BpbZIP4 TF from birch plants that responds strongly to drought stress was obtained. Moreover, plants with overexpressed or RNA-interfered *BpbZIP4* were subjected to evaluate their drought response. In addition, further investigations will be conducted to determine whether *BpbZIP4* can mediate target gene expression through interacting with particular elements in their promoters, thereby influencing plant drought resistance. Our findings will provide a new perspective for breeding new drought-resistant varieties in birch plants.

## Results

### Analysis of conserved domains and phylogenetic tree of BpbZIPs

The domains of 29 birch BpbZIPs proteins were analyzed, including two conserved regions: the basic region and the leucine zipper region (Fig. S1A). The conservation rate of 23 amino acids is >50%, seven amino acids of which have a conservation rate > 90%. Phylogenetic analysis indicates that the 29 birch BpbZIPs and the AtbZIPs of *Arabidopsis* can be classified into 13 subfamilies (Fig. S1B, Table S1: Homologous relationships between birch BpbZIP and *Arabidopsis* AtbZIP TFs). Among them, six BpbZIP TFs of *B. platyphylla*, including BpbZIP4, BpbZIP5, BpbZIP16, BpbZIP19, BpbZIP20, and BpbZIP24, cluster into subfamily A, which is associated with the response to abiotic stress in *Arabidopsis*. This suggests that these six BpbZIP TFs may function as stress-responsive TFs in birch.

### Expression analysis of *BpbZIPs* under drought stress

We subsequently exposed all plants to drought stress via 48 h of continuous water deprivation, over this period the soil water content declined from 33 ± 1.81% to 5 ± 1.04% (Fig. S2A). Reverse transcription quantitative polymerase chain reaction (RT-qPCR) analysis showed that the expression of most BpbZIP subfamily A genes was significantly different under drought stress at various treatment times (6, 9, 12, 24, 48 h) relative to the control (Fig. S2B). Moreover, the expression levels of *BpbZIP4* exhibited significantly higher alterations than those of other *BpbZIP* genes from subfamily A, peaking after 6 h of drought treatment, suggesting that *BpbZIP4* strongly responds to drought stress (Fig. S2B).

### BpbZIP4 is localized in the nucleus

The subcellular localization results showed that the green fluorescence of the 35S:BpbZIP4-GFP fusion protein was observed exclusively in the nucleus, which is consistent with the 4',6-diamidino-2-phenylindole (DAPI) nuclear staining. In contrast, the fluorescence of 35S:GFP was evenly dispersed across the cell, indicating that BpbZIP4 is localized in the nucleus (Fig. S3).

### Characterization of *BpbZIP4* transgenic lines

Antibiotic-resistant birch lines for both *BpbZIP4*-OE and interference expression (RE) were successfully generated (Fig. S4A and B). Three *BpbZIP4*-OE transgenic plants and four interference expression transgenic lines were identified successfully (Fig. S4C). For *BpbZIP4*-OE lines, the expression of *BpbZIP4* ranges from 10 to 55 times the WT (wild type); Line 1 exhibits the highest expression, with Line 2 and Line 3 coming subsequently. For the four RE transgenic lines, *BpbZIP4* expression ranges from 20% to 80% of the WT level. Lines 1 and 2 have the lowest expression (~20%–30% of the control), followed by Lines 3 and 4 (~60% of the control) (Fig. S4D).

### *BpbZIP4* enhances drought resistance in transgenic birch plants

**Figure 1:**
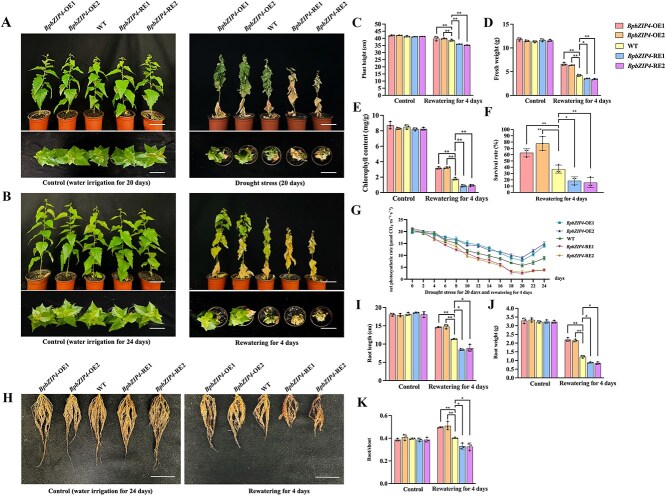
Analysis of the drought resistance of BpbZIP4 transgenic plants. (A–B) Phenotype of *BpbZIP4* transgenic plants in soil under control, 20-day drought stress, and 4-day rewatering. Scale bar represents 10 cm. (C) Plant height. (D) Fresh weight. (E) Chlorophyll content. (F) Survival rate. (G) Net photosynthesis rate. (H) Phenotype of roots of *BpbZIP4* transgenic plants grown in soil under control, drought stress for 20 days, and rewatering for 4 days. Scale bar represents 5 cm. (I) Root length. (J) Root weight. (K) Root/shoot ratio. It refers to the ratio of root weight to the weight of the aboveground part of the plant. Data were presented as means ± standard deviation (SD) of three biological replicates (*n* = 3). * and ** indicate significant differences (*P* < 0.05 and *P* < 0.01).

Under control conditions, all birch plants could grow normally, and there were no significant changes in their height, fresh weight, chlorophyll content, survival rate, and net photosynthetic rates (Figs 1A–G and S5A and B). When exposed to drought stress, *BpbZIP4*-OE lines displayed less leaf wilting than their WT counterparts (Figs 1A and S5C). However, *BpbZIP4*-RE lines showed the opposite situation (Fig. 1A). After a 4-day water recovery, the vegetative growth of *BpbZIP4*-OE lines was more substantially restored, whereas the development of *BpbZIP4*-RE lines showed little recovery (Figs 1B and S5D). In addition, *BpbZIP4*-OE lines showed significant increases of 3.2%, 21.5%, and 29.8% in plant height, fresh weight, and chlorophyll content, respectively, compared with WT plants. In contrast, *BpbZIP4*-RE lines exhibited significant decreases of 7.9%, 17.7%, and 30.3% in these parameters relative to WT plants (Fig. 1C–E). Furthermore, the survival rate of *BpbZIP4*-OE plants was 70%, while that of WT plants was 37%, and the survival rate of *BpbZIP4*-RE plants was only 17% (Fig. 1F); similarly, under continuous drought stress the net photosynthetic rate of *BpbZIP4*-OE lines was 9.46%–64.07% higher than that of the WT, whereas the rate in *BpbZIP4*-RE lines exhibited a reduction of 14.18%–56.49% (Fig. 1G). These findings demonstrated that *BpbZIP4* is capable of boosting drought resistance.

### *BpbZIP4* improves the growth and development of the roots

Under normal growth conditions, all plants exhibited comparable root phenotypes, with no significant variations in root length, weight, or root-to-shoot ratio (Figs 1H–K and S5E). Under 4-day water recovery following drought treatment, compared with WT plants, *BpbZIP4*-OE plants had longer roots, greater root weight, and a higher root-to-shoot ratio, which were significantly higher than those of WT plants by 29.3%, 31.5%, and 24.8%, respectively. Conversely, compared with WT plants, the corresponding traits of *BpbZIP4*-RE lines were notably decreased by 23.5%, 29.4%, and 18.6% respectively (Figs 1H–K and S5F). These findings demonstrated that *BpbZIP4* can promote the growth and development of the roots.

### *BpbZIP4* regulates the histochemical and physiological changes in birch plants

**Figure 2:**
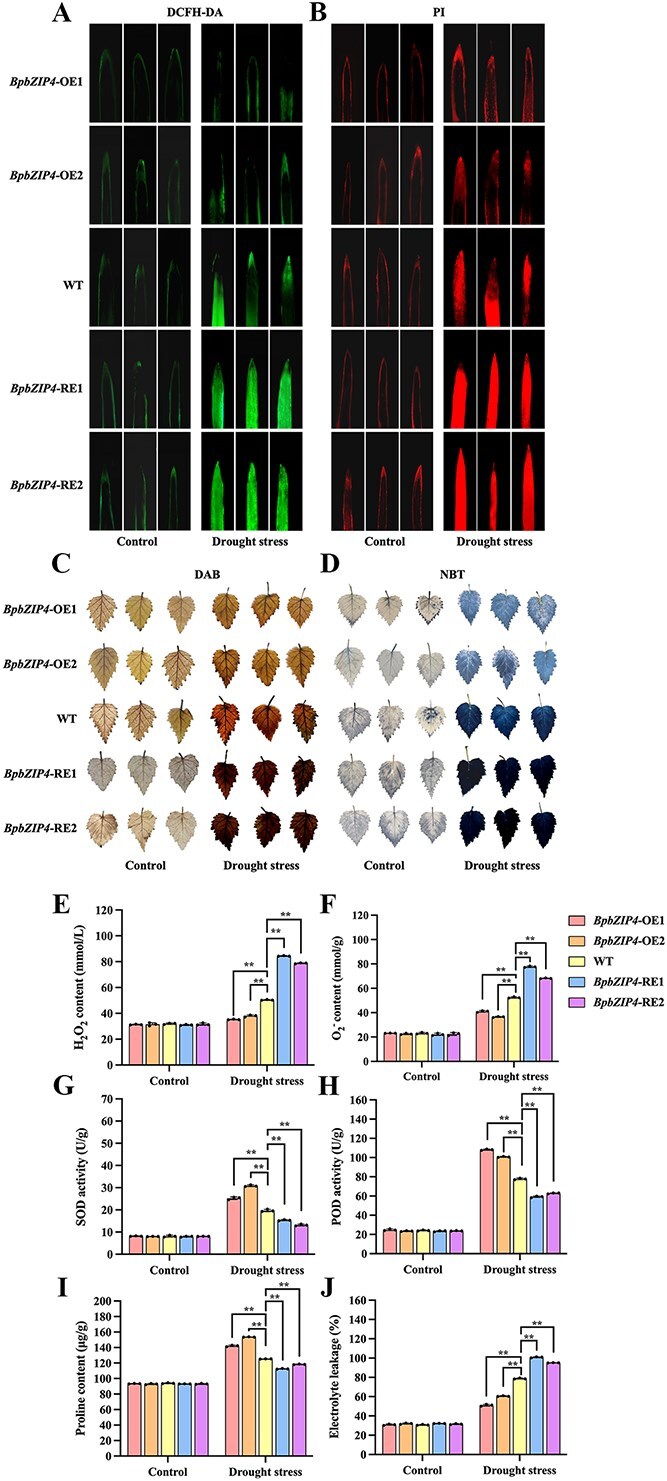
Staining and physiological analysis of BpbZIP4 transgenic plants under drought treatment. (A–D) DCFH-DA, PI, DAB, and NBT stainings. (E–J) Hydrogen peroxide (H_2_O_2_), superoxide anion (O_2_^−^) content, SOD, POD activity, proline content, and electrolyte leakage. Birch seedlings were subjected to drought treatment for 6 h. During this period, well-watered birch plants for 6 h were used as the control group.

The staining and physiological indicators showed no significant differences among plants under control conditions. However, after drought stress, staining in *BpbZIP4*-OE was significantly lighter than in WT plants, while that in *BpbZIP4*-RE was markedly darker (Fig. 2A–D). 2′,7′-Dichlorodihydrofluorescein diacetate (DCFH-DA) (or 3,3′-diaminobenzidine (DAB)) and nitroblue tetrazolium (NBT) staining were employed to assess the accumulation of H_2_O_2_ and O_2_^−^ in the plants. A lighter staining intensity indicates less damage to the plants. These results suggest that *BpbZIP4*-OE is associated with a significantly reduced accumulation of H_2_O_2_ and O_2_^−^, whereas *BpbZIP4*-RE lines accumulate higher levels of these substances (Fig. 2A, C–D). Furthermore, the levels of H_2_O_2_ and O_2_^−^ (Fig. 2E and F) were also consistent with the DCFH-DA (or DAB) and NBT staining results.

Moreover, under drought conditions, the *BpbZIP4*-OE lines exhibited a marked increase in the activities of superoxide dismutase (SOD) and peroxidase (POD), along with a significant rise in proline content. In contrast, the *BpbZIP4*-RE lines showed a distinct decrease in these parameters relative to the WT plants (Fig. 2G–I). Higher SOD and POD activities indicate stronger scavenging ability; higher proline content indicates stronger osmotic adjustment capacity. Additionally, compared to WT plants, Propidium iodide (PI) staining was darker in *BpbZIP4*-OE plants but brighter in *BpbZIP4*-RE plants (Fig. 2B). For electrolyte leakage, levels were lowest in *BpbZIP4*-OE, followed by WT and then *BpbZIP4*-RE plants (Fig. 2J). Both PI staining and electrolyte leakage reflect plasma membrane and cell membrane damages: lighter staining and lower leakage indicate less severe damage. These results demonstrate that *BpbZIP4* is capable of boosting plants’ ability to scavenge ROS and of mitigating cellular damage.

### *BpbZIP4* influences stomatal opening and water loss

**Figure 3:**
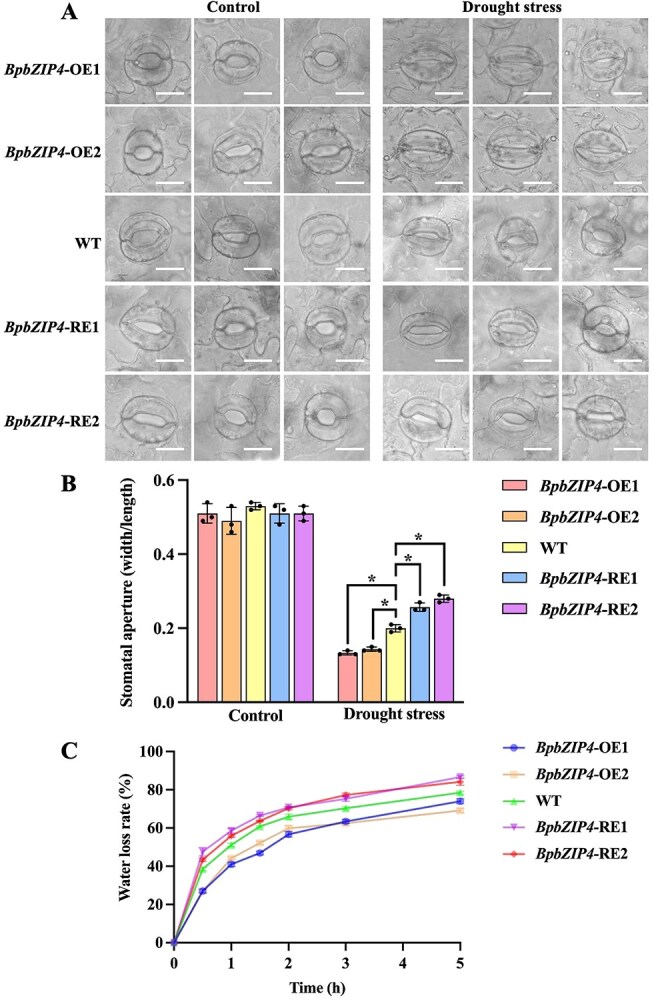
Analysis of stomata and water loss rate of BpbZIP4 transgenic lines during drought treatment. (A) Stomatal phenotype. Scale bar = 20 μm. (B) Stomatal apertures measured from three randomly selected stomata in each experimental replicate. (C) Water loss rates measured from six leaves randomly selected in each biological replicate.

Under control conditions, stomatal size did not differ significantly in all the plants. However, under drought stress, the *BpbZIP4*-OE lines exhibited significantly smaller stomata, along with a notable reduction in the ratio of stomatal width to length. In contrast, the *BpbZIP4*-RE birch plants displayed an opposite trend (Fig. 3A and B).

Stomatal opening and closing are essential for regulating water transpiration in plants. We assessed the water loss rates from the leaves of *BpbZIP4* transgenic birch plants and WT birch plants. The findings reveal that the *BpbZIP4*-OE plants exhibited a lower water loss rate compared to the WT plants over time, whereas the *BpbZIP4*-RE plants displayed a higher one (Fig. 3C).

### RNA sequencing analysis

To determine the role of *BpbZIP4* in drought regulation, the target genes of *BpbZIP4* were identified by RNA sequencing (RNA-seq). Under drought stress, comparative analyses of birch showed 8683 differentially expressed genes (DEGs) between the *BpbZIP4*-OE lines and WT (6082 upregulated, 2601 downregulated), and 6768 DEGs between the *BpbZIP4*-RE lines and WT (4819 upregulated, 1949 downregulated) (Fig. S6A). Moreover, *BpbZIP4*-OE lines had the most DEGs, while those of *BpbZIP4*-RE had the least DEGs, and 1475 coexpressed genes were differentially expressed in the *BpbZIP4*-OE, RE, and WT plants (Fig. S6B).

### Establishment and verification of the GRN based on BpbZIP4

**Figure 4:**
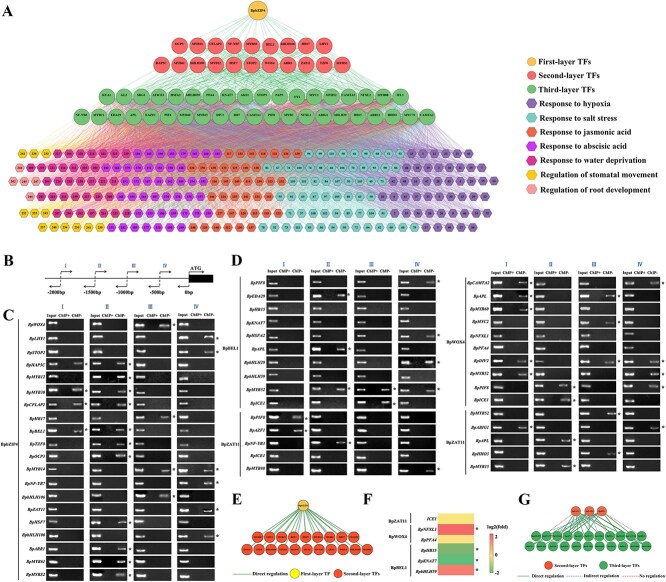
Construction of drought-stressed GRN in *B. platyphylla* and verification of layer 1–3 gene regulatory relationships. (A) The GRN of *B. platyphylla* constructed based on *BpbZIP4* transgenic plants under drought treatment. The top layer is the BpbZIP4 TF, the second has 20 TFs, the third 39 TFs, and the fourth 247 structural genes associated with seven enriched GO biological processes. This GRN was constructed under drought treatment for 6 h. (B–G) Verification of the gene regulatory relationships from the first to the third layer in the GRN. (B) The promoter of the target genes (−2000 to 0 bp) was divided into four equal-length fragments for ChIP-PCR. (C–D) ChIP-PCR validation of gene regulatory relationships between layers 1st–2nd and 2nd–3rd. Input: the chromatin before IP. ChIP+: IP with anti-FLAG antibody. ChIP–: IP with no antibody. The asterisk (*) represents that the TFs can bind to the promoters of the target genes. (E) A schematic diagram of the gene regulatory relationships between the first and second layers (F) RT-qPCR validation of regulatory relationships between layers 2nd–3rd. The asterisk (*) represents significant regulation (fold change ≥2; *P* < 0.05). (G) A schematic representation of the gene regulatory associations between the second and third layers.

A four-layer GRN was established, with the top layer containing the BpbZIP4 TF, the second layer comprising 20 TFs, the third layer 39 TFs, and the fourth layer 247 structural genes (Tables S2 and S3: The layer assignment of each gene from first to third in the GRN; Characteristics of the genes in the fourth layer of the GRN). These genes are primarily enriched in the seven gene ontology (GO) pathways related to stress response, including the response to hypoxia, salt stress, jasmonic acid, ABA, water deprivation, and regulation of stomatal movement and root development (Fig. 4A). When plants are subjected to drought stress, the GRN comprises 4778 forecasted relationships—20 linking the BpbZIP4 TF to second-layer TFs, 332 connecting second- and third-layer TFs, and 4426 bridging third-layer TFs and fourth-layer structural genes. Regulatory signals from the top layer were transmitted to second-layer and third-layer TFs and further passed down to fourth-layer genes, which then execute specific functions in drought stress responses.

To investigate the regulatory interactions between genes in GRN, some TFs from different layers were randomly selected. The truncated promoters (2000 bp in length) of the putative target genes were partitioned into four equal regions (Fig. 4B). ChIP-PCR assay results illustrated that all 20 interactions between BpbZIP4 TF (first layer) and the promoters of second-layer TFs were confirmed. This outcome demonstrates that 100% of the regulatory relationships are direct regulations (Fig. 4C and E). Concerning the regulatory interactions between the second and third layers within the GRN, ChIP-PCR results demonstrated that 24 of the 30 interactions were direct regulatory ones (Fig. 4D), while the remaining six exhibited no evidence supporting direct regulation. RT-qPCR analyses indicated indirect regulation in four of the six interactions, with the remaining two showing no significant interaction-related changes (Fig. 4F). These data revealed 80% of the 30 interactions were direct regulations and 13% were indirect, meaning 93% of the predicted regulatory relationships between the second and third layers were confirmed overall (Fig. 4G). The same method was applied to verify the regulatory relationships between genes in the third and fourth layers. ChIP-PCR results illustrated that 25 out of the 30 relationships were direct regulatory interactions (Fig. S7A and B). RT-qPCR assay outcomes indicated two indirect regulatory relationships, with three others displaying no significant regulatory associations (Fig. S7C). The combined data confirmed that 90% of the predicted regulatory relationships between the third and fourth layers were verified overall (Fig. S7D). These data demonstrated that the regulatory relationships in the GRN are reliable.

### Target gene expression profiles during drought stress

**Figure 5:**
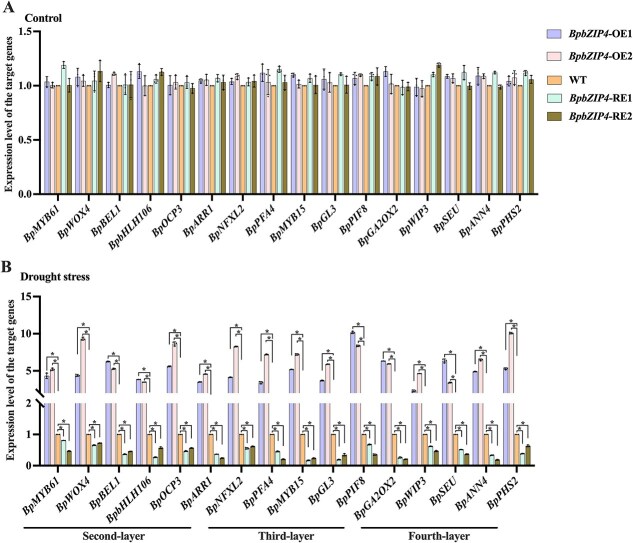
The expression levels of the target genes from the second to fourth layers in the GRN in *BpbZIP4* transgenic plants under drought treatment. (A–B) Expression levels of target genes in *BpbZIP4* transgenic plants and WT plants under both control and 6-h drought treatment. Data are presented as means ± SD of three biological replicates (*n* = 3).

The expression profiles of some target genes randomly selected from the GRN’s second to fourth layers were analyzed in *BpbZIP4* transgenic lines under drought treatment for 6 h. Results showed that these genes exhibited no significant expression differences when plants were under control conditions (Fig. 5A). However, following drought stress, these genes were expressed at significantly higher levels in *BpbZIP4*-OE transgenic birch than in WT plants, but at lower levels in *BpbZIP4*-RE plants (Fig. 5B). The results illustrate that *BpbZIP4* is capable of modulating the expression of these GRN genes during drought conditions.

### Identifying the specific *cis*-acting elements of BpbZIP4

TF-centered Y1H results indicated that BpbZIP4 is capable of binding one known element GATA-box (ATGATAAGG), and two unknown novel elements (GTGGTGG and GGGGATG) (Fig. S8A and B). To define the central sequences of the two unknown new elements, DNA base deletions were performed. Experimental results illustrated that the third base from the left (‘T’) and the third base from the right (‘T’) of new element 1 are necessary for binding (Fig. S8C). For new element 2, the third base from the left (‘G’) and the second base from the right (‘T’) are key to binding activity (Fig. S8D). Therefore, the central sequences of two new elements were confirmed as (‘TGGT’) and (‘GGAT’), which were named TGGT-box and GGAT-box, respectively. Additionally, base mutation results indicate that in the core sequence of element 1, the first base (‘T’) can be replaced with ‘C’, whereas the other bases cannot be substituted; none of the bases in novel element 2 can be replaced by any other base (Fig. S8E and F). Our findings revealed that the conserved sequences bound by BpbZIP4 were ‘[C/T]GGT’ for new element 1 and ‘GGAT’ for new element 2. These results demonstrate that BpbZIP4 interacts with three specific elements, including the GATA-box, TGGT-box, and GGAT-box.

### Interaction between BpbZIP4 and target gene promoters

**Figure 6:**
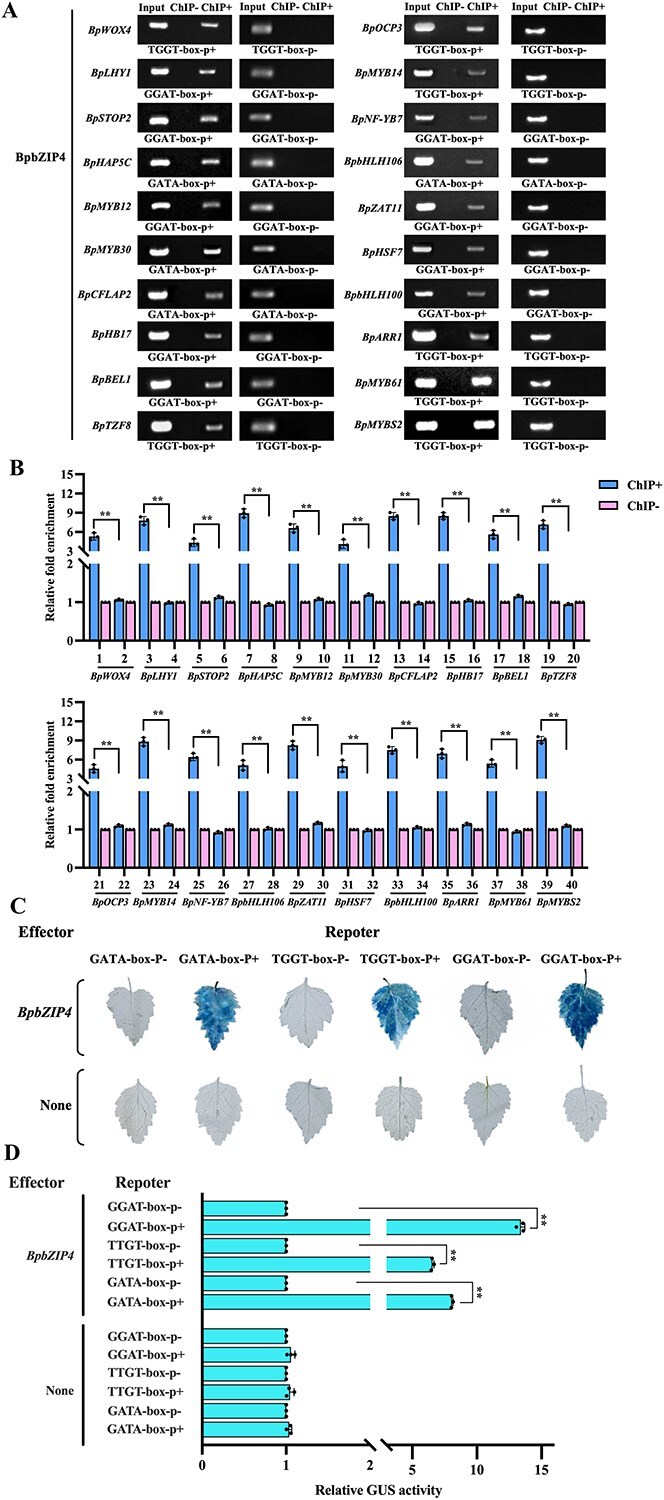
Confirming the binding of BpbZIP4 to target gene promoters from the GRN via ChIP and GUS assays. (A–B) Establishing direct interplays of BpbZIP4 with second-layer TFs via ChIP-PCR and ChIP-qPCR. TGGT-box- p+/p–, GGAT-box- p+/p–, and GATA-box- p+/p–: a shortened promoter with or without the TGGT, GGAT, and GATA element. 1, 2, 17–26, 37–40: the truncated promoters featuring or missing the TGGT; 3–6, 9,10, 15, 16, 25, 26, 29–34: the truncated promoters featuring or missing the GGAT; 7, 8, 11–14, 27, 28: the truncated promoters featuring or missing the GATA. The relative fold enrichment in ChIP– samples were set to 1. (C–D) Analysis of GUS staining and activity for BpbZIP4 interaction with target gene promoters via specific element binding.

The 35S:BpbZIP4-Flag was introduced into birch via transformation, followed by ChIP analysis to capture BpbZIP4-bound promoter fragments of second-layer genes in the GRN. Truncated target gene promoters containing two new elements (TGGT-box and GGAT-box) alongside one known element (GATA-box) were isolated via ChIP utilizing a Flag antibody. ChIP-PCR indicated marked levels of truncated promoters harboring these three elements in Flag antibody-treated samples, whereas those lacking the elements showed no enrichment (Fig. 6A). Moreover, ChIP-qPCR results showed that the abundance of truncated promoters containing the TGGT, GGAT, and GATA elements in the ChIP+ group was 3- to 10-fold higher than that in the ChIP– group, with ChIP– set as 1 (Fig. 6B).

Furthermore, β-glucuronidase (GUS) staining showed a significantly greater intensity of BpbZIP4 interaction with the TGGT-box, GGAT-box, and GATA-box in contrast to control samples (Fig. 6C). Additionally, transformant lines harboring these three elements exhibited a notable rise in GUS activity, with elevations of roughly 5- to 14-fold relative to control samples (Fig. 6D). These results reveal that BpbZIP4 has the ability to bind to truncated target gene promoters containing the three specific elements.

### Target genes regulated by *BpbZIP4* mediate drought resistance

Six TFs from the second layer, including BpHB17, BpbHLH100, BpWOX4, BpBEL1, BpZAT11, and BpMYB61 (GenBank number: PX523769-PX523773, OP490311) were selected randomly. They are related to response to ABA, salt stress, water deprivation, regulation of root development, and stomatal movement (Table S4: Characteristics of the 6 second-layer TFs under 6 h drought stress in the GRN). Among these six genes, the expression levels of *BpMYB61*, *BpWOX4*, and *BpBEL1* were significantly upregulated, while those of other three genes were significantly downregulated, based on the RNA-seq data.

The stomatal aperture was measured in six OE plants and pCMBIA1307 empty vector plants. When subjected to control conditions, all plants exhibited no significant discrepancies (Fig. S9A and B). However, after drought stress, stomatal aperture in *BpMYB61*-OE, *BpWOX4*-OE, and *BpBEL1*-OE plants significantly decreased compared with pCMBIA1307 empty vector plants, while *BpbHLH100*-OE, *BpHB17*-OE, and *BpZAT11*-OE exhibited an increase (Fig. S9A and B). These results indicated that under drought stress conditions, *BpMYB61*, *BpWOX4*, and *BpBEL1* can reduce stomatal opening, while the other three genes can increase it.

**Figure 7:**
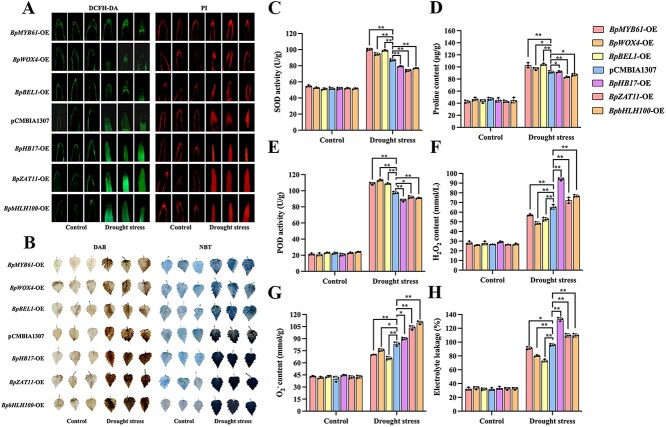
Staining analysis in transgenic birch plants harboring six GRN genes under drought treatment. (A–B) DCFH-DA and PI, DAB, and NBT stainings. (C) SOD activity. (D) Proline level. (E) POD activity. (F) Hydrogen peroxide (H_2_O_2_) content analysis. (G) Superoxide anion (O_2_^−^) content analysis. (H) Electrolyte leakage analysis. Data were presented as means ± SD of three biological replicates (*n* = 3).

Additionally, the stainings and physiological indicators were analyzed. When subjected to control conditions, no meaningful differences were observed across all plants. Under drought stress, *BpMYB61*-OE, *BpWOX4*-OE, and *BpBEL1*-OE plants exhibited lower DCFH-DA and PI fluorescence intensity and lighter DAB and NBT stainings. However, *BpbHLH100*-OE, *BpHB7*-OE, and *BpZAT11*-OE plants displayed the opposite trend (Fig. 7A and B). Furthermore, compared with pCMBIA1307 plants, *BpMYB61*-OE, *BpWOX4*-OE, and *BpBEL1*-OE plants showed higher SOD, proline levels and POD, lower H_2_O_2_, O_2_^−^, and electrolyte leakage. In contrast, the corresponding indicators in *BpbHLH100*-OE, *BpHB17*-OE, and *BpZAT11*-OE plants exhibited the opposite trend (Fig. 7C and H). The above results indicate that BpMYB61, BpWOX4, and BpBEL1 can enhance plant drought resistance, while the other three genes exert the reverse effect.

## Discussion

Acting as crucial regulators in how plants respond to abiotic stresses, bZIP TFs have had their functions validated in a range of species including *Oryza sativa*, *Arabidopsis thaliana*, *Glycine max*, and *Zea mays* [20–23]. However, research into the mechanisms by which bZIP mediates drought resistance in trees remains unclear. In this study, we cloned the birch *BpbZIP4* gene and investigated its role in drought stress responses. Further analysis revealed that BpbZIP4 binds to specific elements in truncated promoters, thereby regulating target gene expression within the GRN. Additionally, the function of key TFs from the GRN during drought stress was demonstrated. These findings reveal that BpbZIP4 transmits stress signals layer by layer by regulating the expression of target genes in the GRN, thereby enhancing the drought resistance of birch.

### BpbZIP4 enhances drought resilience by improving the root growth

Root growth is crucial for shaping the overall root system architecture, supporting plant adaptation to abiotic stress conditions [24–26]. Some studies have shown that bZIP TFs can influence plants’ stress tolerance by regulating root growth and development. For instance, *ZmbZIP4* enhances abiotic stress resistance in *Z. mays* (maize) by regulating lateral root number, extending primary root length, and optimizing the root system [27]. *ZmbZIP89* in maize regulates the homeostasis of ROS in roots, thereby increasing lateral root length and enhancing drought resistance in plants [28]. In this study, *BpbZIP4*-OE birch plants exhibited less leaf wilting and a higher survival rate, indicating that *BpbZIP4* enhances birch’s drought tolerance (Fig. 1A–G). Furthermore, *BpbZIP4*-OE lines were found to increase root length, root weight, and root-to-shoot ratio under drought conditions (Fig. 1H–K). These results demonstrate that *BpbZIP4* enhances drought tolerance in birch by regulating root growth.

### BpbZIP4 regulates target gene expression by specifically interacting with the elements

Transcription factors modulate target gene expression by attaching to promoter elements [29]. Research has shown that bZIP TFs mainly control target gene expression via binding to ABRE (‘ACGTGTC’) and G-box (‘CACGTG’) elements under drought, salt, heat, and cold stress conditions [30–32]. Furthermore, a bZIP5 TF from *B. platyphylla* regulates the expression of genes related to plant tolerance to saline–alkali stress by binding to four novel elements: GGGG-box (ATGGGGC), GATC-box (GGGATCG), CTAC-box (CCTACAA), and CCGA-box (GACCGAA). Nonetheless, it stays unknown if bZIP TFs bind to distinct *cis*-acting elements and/or novel elements when responding to diverse biological pathways. This research employed TF-centered Y1H, ChIP, and GUS analyses, which demonstrated that BpbZIP4 controls the expression of genes related to drought stress responses via its interaction with a photosynthesis-related GATA-box element and two novel elements (TGGT-box and GGAT-box) (Figs S8 and 6). Our finding provides a novel perspective for elucidating the response mechanism of bZIP TFs under drought stress.

### BpbZIP4 as the key regulator in the GRN for drought resistance

To cope with the harmful impacts of abiotic stress, plants have developed diverse response systems that involve numerous genes and thus form a complex GRN. In a GRN, top-layer TFs are pivotal regulators with core significance [33]. Thus, constructing GRNs is critical for identifying key genes involved in stress responses. For example, a four-layer GRN under cadmium treatment was constructed in *Tamarix hispida*, which identified important top-level TFs associated with cadmium stress [34]. Moreover, in *B. platyphylla*, a GRN under drought stress was built, and the found top TFs enhance drought resistance by regulating multiple pathways [35]. In this study, phenotypic, staining, and physiological index results demonstrated that *BpbZIP4* enhances drought tolerance in *BpbZIP4*-OE birch lines (Figs 1 and 2). Additionally, a four-layer drought-responsive GRN was constructed in *B. platyphylla* based on *BpbZIP4* transgenic lines (Fig. 4). As a top-layer regulator, BpbZIP4 plays a central role in enhancing drought resistance.

The top-layer GRN TFs transmit stress signals to second-layer TFs, which relay them hierarchically to induce expression changes of structural genes. These alter multiple biological pathways, influencing stress tolerance in plant species [36]. In our research, BpbZIP4 modulates the expression of second-layer TFs through its interaction with specific promoter elements (Fig. 6). Furthermore, functional studies on second-layer TFs in the GRN revealed that they significantly affect the drought resistance of birch, demonstrating that these TFs play key roles (Figs 7 and S9). On the whole, this study confirms that *BpbZIP4* can enhance the drought tolerance of birch by regulating the expression of downstream genes.

Moreover, it is currently not yet established whether bZIP TFs exhibit other regulatory mechanisms governing stress resistance. A study revealed that the rice TF OsbZIP72 interacts with the OsGSK1 protein to inhibit the expression of the downstream gene OsNHX1, thereby enhancing the salt tolerance of rice [28]. In peppers, the bZIP TF CaDRHB1 modulates the ABA signaling pathway and the expression of stress-responsive genes through small ubiquitin-like modifier modification (SUMO), thus improving the drought tolerance of peppers [37]. However, it remains unclear whether BpbZIP4 influences birch’s abiotic stress resistance through interactions with other proteins or its own post-translational modifications (PTMs). Further exploration into these regulatory mechanisms is required.

## Conclusion

In brief, an operational model was constructed for BpbZIP4 as the top regulator in the four-layer GRN under drought treatment (Fig. S10). When exposed to drought stress, *BpbZIP4* expression is triggered, and it modulates second-layer GRN TFs via association with three particular elements in their promoters. These TFs then relay hierarchically to govern expression changes from third-layer TFs to fourth-layer structural genes. This regulatory cascade triggers changes in seven biological pathways, including responses to hypoxia, salt stress, jasmonic acid, ABA, water deprivation, regulation of stomatal movement, and root development, thereby enhancing birch drought resistance.

## Materials and methods

### Plant material and drought treatment

Birch seeds were placed in the soil. The conditions in the growth chamber feature a 16/8 h day/night period, with a temperature of 25°C and a humidity of 65%–70%. Subsequently, 2-month-old plants were exposed to natural drought treatment for 6, 9, 12, 24, and 48 h, while the control group was watered normally for 48 h. During drought treatment, soil water content (SWC, v/v) of all groups was continuously monitored with a Takeme10 portable soil moisture sensor (Takeme, Beijing, China). The samples were then harvested. Three biological repetitions were conducted.

### Domain structure and construction of phylogenetic tree

The coding sequences (CDS) of all BpbZIPs were retrieved from the *B. platyphylla* genome. The structural domains of BpbZIPs were examined via an online website resource: http://weblogo.berkeley.edu/logo.cgi. A phylogenetic tree encompassing *B. platyphylla*’s BpbZIP proteins and all *Arabidopsis* AtbZIP proteins was constructed using MEGA 7.0. This analysis included 1000 bootstrap replications based on the *P*-distance model.

### The expression assessment of the genes

RNA of birch plants was extracted with an RNA extraction kit (OMEGA, Norcross, USA). Afterward, the protocols for reverse transcription and qPCR were followed according to the published paper [38,39]. The levels of gene expression were analyzed, including six bZIP TFs (GenBank numbers: OR727895, PV693704, PX523765-PX523768) from the A subfamily and the target genes regulated by *BpbZIP4* in *B. platyphylla*. Primers used are recorded in Table S5: Primer sequences for qRT-PCR analysis.

### Subcellular distribution of BpbZIP4

The cloned CDS of *BpbZIP4* was inserted into the pBI121-GFP vector, and this recombinant vector was then delivered into onion cells via transformation. The empty GFP vector was used as a reference control. After 48 h had elapsed, the cells were treated using DAPI (100 ng/ml) and then examined. Primers used are provided in Table S6: Primer sequences of BpbZIP4 constructed into pBI121-GFP.

### Creation of *BpbZIP4-*overexpressing and interfering transgenic birch plants

The coding sequence of *BpbZIP4* underwent PCR amplification and was subsequently introduced into the pROKII vector to obtain its OE. A shortened form of *BpbZIP4* that included both sense and antisense sequences was inserted into a modified pROKII vector for RNA interference (RE). These constructs were introduced into *Agrobacterium tumefaciens* EHA105, and were transformed into birch plants by *A. tumefaciens*-mediated method [40].

The plants were cultivated in a selection medium containing 50 mg/l kanamycin. The medium of birch tissue culture for different growth stages is detailed in previously published papers [41]. DNA was isolated, positive transgenic lines were identified, and total RNA was isolated. Subsequently, the expression abundances of *BpbZIP4* were analyzed. Primers used are provided in Table S7: Primer sequences for constructing the *BpbZIP4*-OE and RE vectors.

### Phenotypic investigation of *BpbZIP4* transgenic plants during drought exposure

After 45 days of growth in soil, BpbZIP4 transgenic birch plants and their WT counterparts were subjected to a 20-day natural drought period induced by water deprivation. Following the drought treatment, the birch plants were rewatered for 4 days. Throughout these days, plants receiving adequate water supply functioned as the control. Subsequently, the plant height, fresh weight, survival rate, root length, and root/shoot ratio were measured. The chlorophyll content was analyzed according to methods as depicted earlier [42]. For 24 consecutive days, leaf net photosynthesis rate was evaluated using a handheld photosynthesis analyzer. Stomates on the leaf epidermis were observed, and their width-to-length ratios were determined using an inverted fluorescence microscope. The rate of water loss was assessed during dehydration treatment, following the outlined methodology [43].

### Histochemical and physiological analysis

Root tips were stained with DCFH-DA and PI, in accordance with the outlined protocol [44, 45]. The staining protocols for NBT and DAB adhered to the previous report [46]. The activities of SOD and POD, hydrogen peroxide (H_2_O_2_) and Superoxide anion (O_2_^−^) concentrations were evaluated by Assay Kit (Nanjing Jiancheng Bioengineering, Nanjing, China). The proline levels, electrolyte leakage, and MDA content were evaluated based on previous research [47–49].

### RNA sequencing

BpbZIP4 transgenic lines and WT plantlets were cultivated in soil under the same conditions for 30 days, then exposed to natural drought conditions for 6 h. Subsequently, leaves from the plants were collected, with three biological replicates. The cDNA libraries were prepared through mRNA enrichment and RT-PCR, and sequencing was performed using the Illumina (Illumina, San Diego, USA).

Differential expression analysis of RNA between the treatment and control groups was carried out using DESeq2 software [50], while edgeR software was applied for analyzing differential expression between these two sample sets [51]. Genes were regarded as DEGs when they satisfied the criteria of a false discovery rate (FDR) < 0.05 and an absolute fold change ≥2 [52]. Gene analysis was carried out using BLASTX databases. To identify biological processes that are overrepresented in birch under drought stress, GO assessment was carried out targeting the DEGs.

### Construction of the GRN under drought stress and validation

Additionally, following the computational approach outlined in a prior study [33], a TF-driven GRN was constructed using an algorithm that relies on partial correlation coefficients and targets DEGs regulated by BpbZIP4. The process involves identifying pairs of coexpressed genes together with a TF. Coexpressed structural genes were identified using thresholds of a correlation coefficient (CC) ≥ 0.8 and a *P*-value <0.001 [53]. The partial correlation coefficient (PCC) for coexpressed gene pairs was calculated using the algorithm r_xy|z=_$\frac{{\mathrm{r}}_{\mathrm{xy}}-{\mathrm{r}}_{\mathrm{xy}}{\mathrm{r}}_{\mathrm{yz}}}{\sqrt{1-{\mathrm{r}}_{\mathrm{xz}}^2}\sqrt{1-{\mathrm{r}}_{\mathrm{yz}}^2}}$. In this equation, x and y represent the coexpressed gene pair, and z represents a TF. A PCC value <0.3 for the coexpressed gene pair was considered significant [54].

Moreover, to verify interlayer gene regulatory relationships, this study conducted ChIP-PCR and RT-qPCR assays. The specific procedures are as follows: some TFs were randomly selected from the second and third layers to construct the pCAMBIA1307–35S::TF-3 × FLAG vector, which was introduced into birch via transient transformation [55]. Transgenic plants were harvested; ChIP assays were done with a FLAG antibody as described [56], followed by ChIP-PCR to confirm direct regulatory interactions between genes of different layers. Additionally, RNA from transgenic plants was extracted, reverse-transcribed into cDNA, and RT-qPCR was used to detect gene expression and confirm indirect regulatory relationships. Primers for both assays are in Table S8: Primer sequences used for the verification of the GRN.

### TF-centered Y1H

A DNA random insertion library was generated and introduced into Y187, which functioned as the reporter, as described in a prior study [36]. Employing TF-centered Y1H technology [36], the pGADT7-Rec2 vector was engineered to contain BpbZIP4 (serving as effector) and then introduced into Y187, which contains the reporter system. Next, the cells were cultured for 3–5 days at 30°C on SD/-Trp/-Leu/-His (TDO) medium containing 50 mM 3-AT (3-amino-1,2,4-triazole) to screen for positive colonies. Next, the cells were cultured for 3–5 days at 30°C on SD/-Trp/-Leu/-His (TDO) growth medium containing 50 mM 3-AT (3-amino-1,2,4-triazole) to screen for positive colonies. Following this, plasmids were extracted, transformed into Top 10 competent cell, and subsequently sequenced.

To further validate the central sequences of the new elements, portions of the base sequences were removed from the adjacent segments on both the left and right sides. Subsequently, the two core sequences were mutated by substituting ‘A/T’ bases with ‘C’ and ‘G/C’ bases with ‘A’. Three consecutive repetitions of these mutated DNA sequence fragments were constructed into pHIS2 as reporter vectors. Each reporter vector, together with pGADT7-Rec2-BpbZIP4, was cotransformed into yeast strain Y187. Primers used are provided in Table S9: Primer sequences used for TF-centered Y1H and Y1H.

### ChIP assay

BpbZIP4 fused with a Flag sequence was cloned into the pROKII vector, then transformed into EHA105. Whereafter, it was delivered into birch plantlets using a transient transformation method. ChIP assays were performed via a Flag antibody according to the procedure [56]. The sonicated supernatant served as input. ChIP was conducted using the Flag antibody for the ChIP+ sample, while the ChIP– sample lacked antibody. ChIP-PCR and ChIP-qPCR were used to assess target genes in the second layer of the BpbZIP4-regulated GRN, following a previously published protocol [35]. Primers used in this study can be found in Table S10: Primer sequences used for ChIP assays. The positions of the short DNA fragments with and without the specific element on the promoter of the target gene are shown in Fig. S11A.

### GUS analysis

Truncated promoter fragments of the second-layer TFs in the GRN—both with and without the specific element—were selected and fused to the GUS gene to serve as reporters (Fig. S11B). Each reporter construct was transiently transformed into *BpbZIP4*-overexpressing (*BpbZIP4*-OE) birch in accordance with a public procedure [55]. Birch leaf samples underwent GUS staining following the public protocol [57]. Subsequent quantification of GUS activity was performed utilizing the Plant GUS Elisa Kit (Yuanju Biotechnology, Shanghai, China). Primers used are provided in Table S11: Primer sequences utilized for GUS assay.

### Assessment of drought resistance of target genes regulated by BpbZIP4

The CDS of each TF from the second layer of the GRN was ligated into the pCAMBIA1307-FLAG vector. These vectors were then transiently transformed into *B. platyphylla* plants using the method reported by [55]. Birch plants transiently transformed with the empty pCAMBIA1307–3 × FLAG vector served as controls. Thereafter, drought stress was applied to the plants by means of a 20% PEG 6000 solution, with the treatment lasting 6 h. Subsequently, DCFH-DA, PI, DAB, and NBT staining was conducted, alongside measurements of SOD and POD activities, proline content, electrolyte leakage, and the levels of H_2_O_2_ and O_2_^−^. Primers used are detailed in Table S12: Primer sequences used for constructing pCMBIA307-Flag-TFs in the GRN.

### Statistical assessment

The statistical assessment was executed by means of SPSS 22 (IBM Corp., Armonk, NY, USA). Differences among multiple groups were evaluated using one-way analysis of variance (ANOVA); if a significant overall effect was observed (*P* < 0.05), *post hoc* comparisons were further conducted using Tukey’s honest significant difference (HSD) test to identify specific pairwise differences. One and two asterisks (* and **) display a significant difference (*P* < 0.05 and *P* < 0.01).

## Supplementary Material

Web_Material_uhag002

## Data Availability

All relevant data are presented within the paper and its supplementary files. The RNA-Seq raw data can be found at NCBI (PRJNA1363668).
