# Supplementary figures and images for: A BpbZIP4 transcription factor enhances drought resistance and root development in Betula platyphylla: insights into a gene regulatory network
Source: Hortic Res. 2026 Jan 7;13(4):uhag002. doi: 10.1093/hr/uhag002 (PMC13098369; doi:10.1093/hr/uhag002)

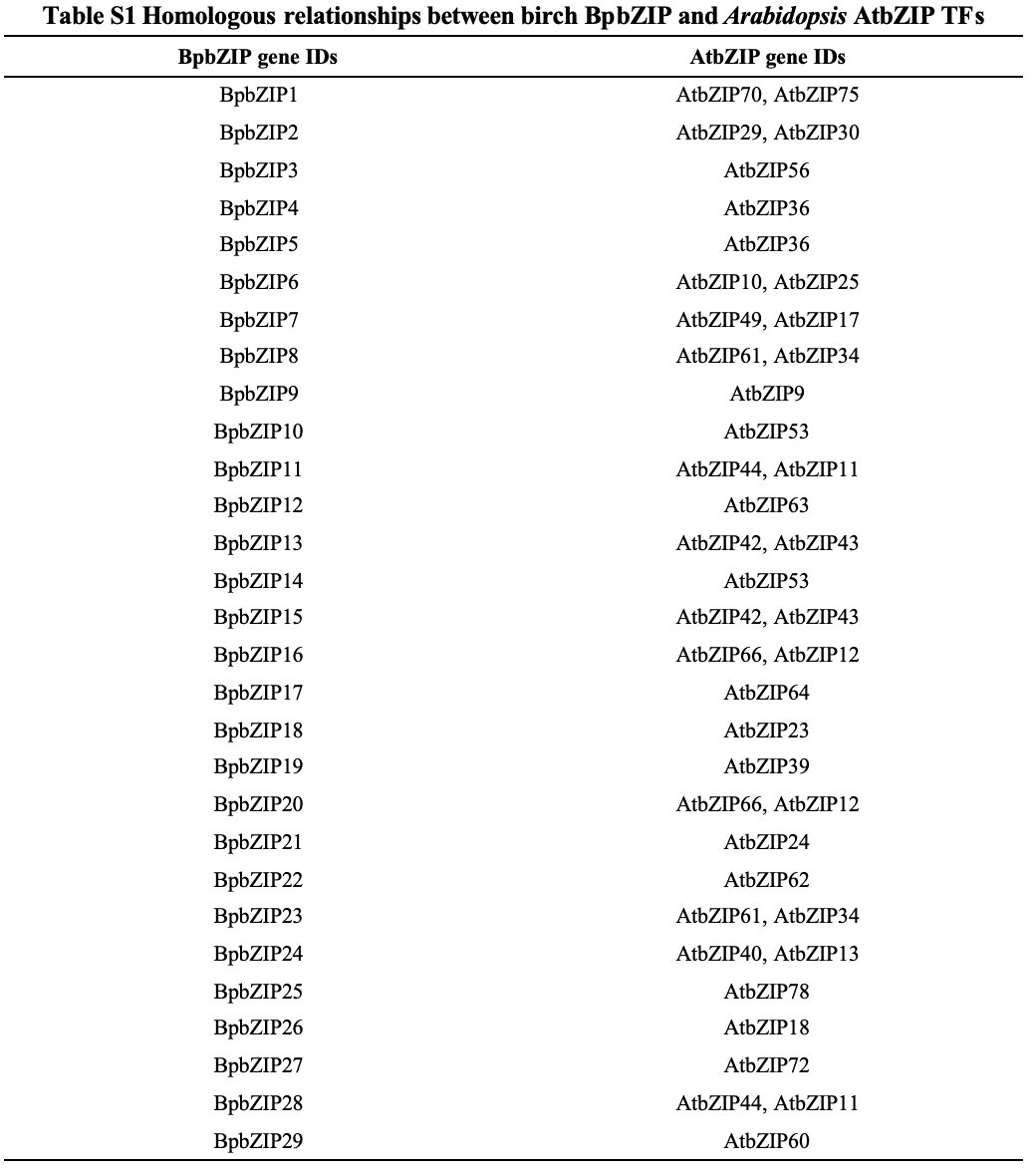

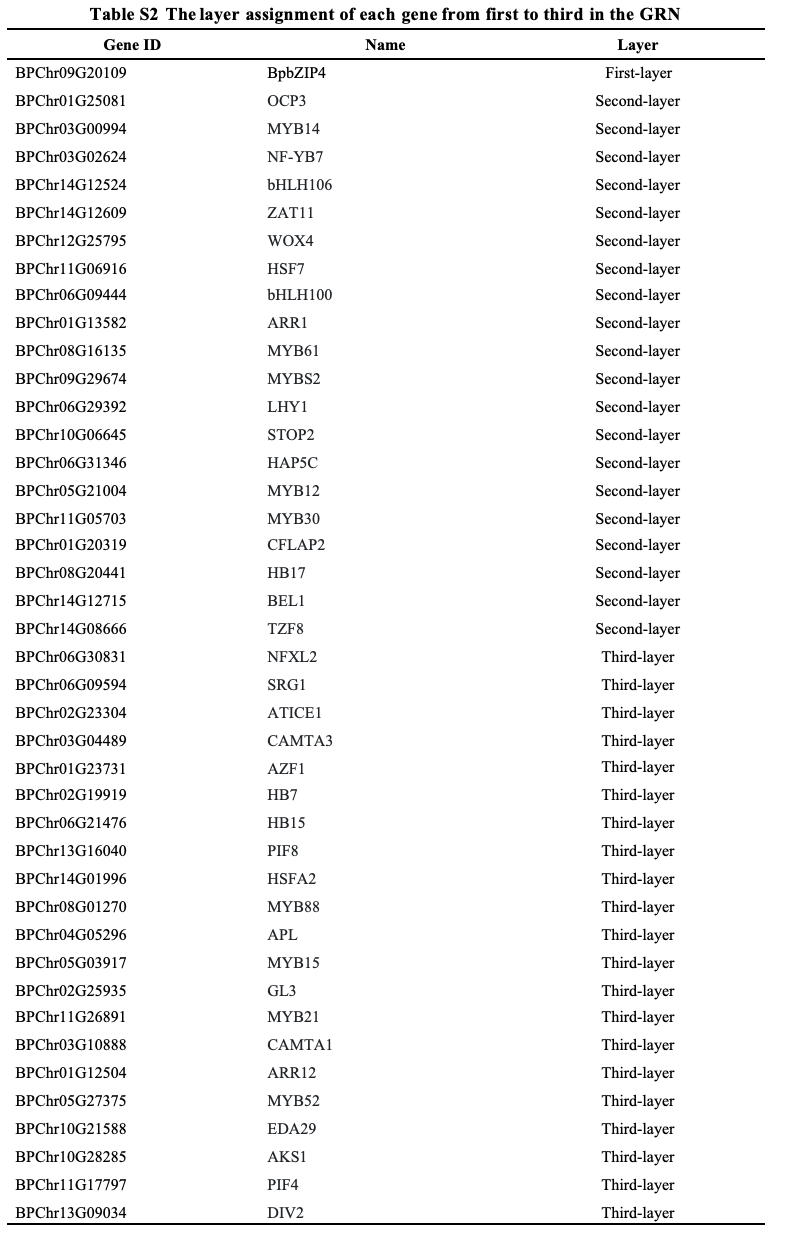

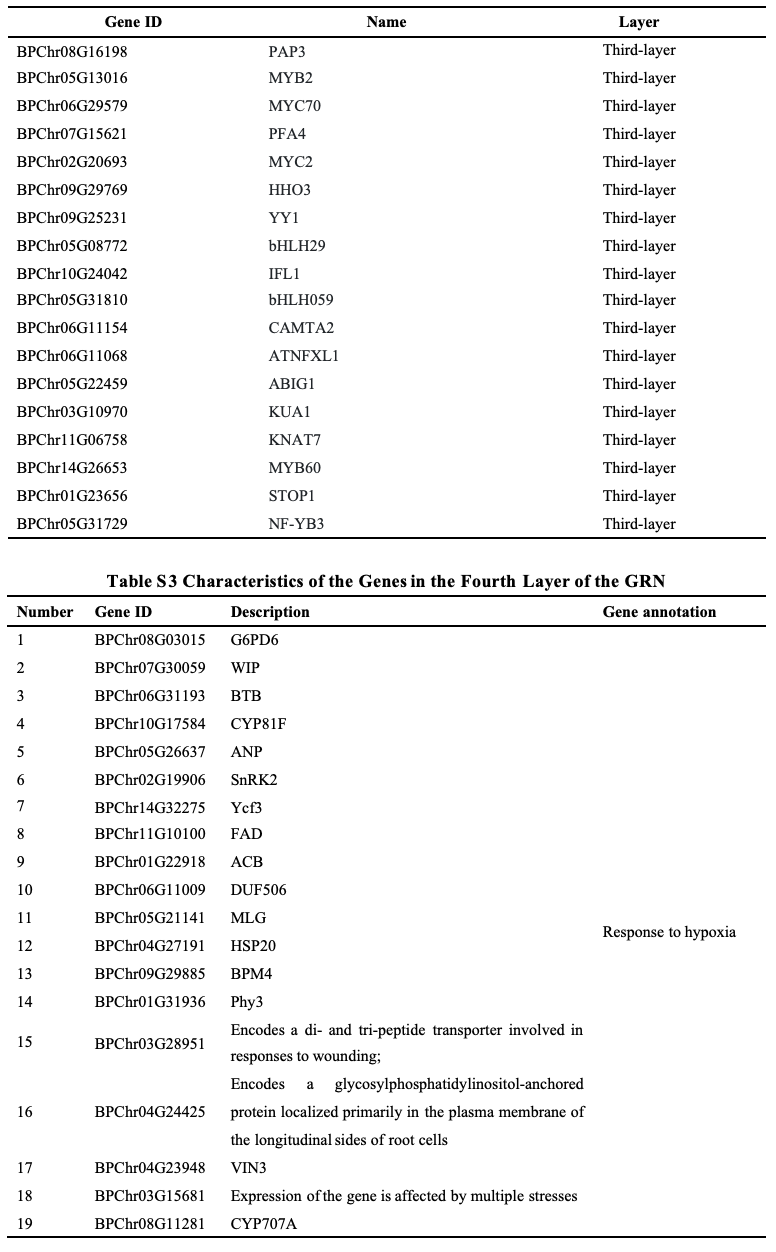

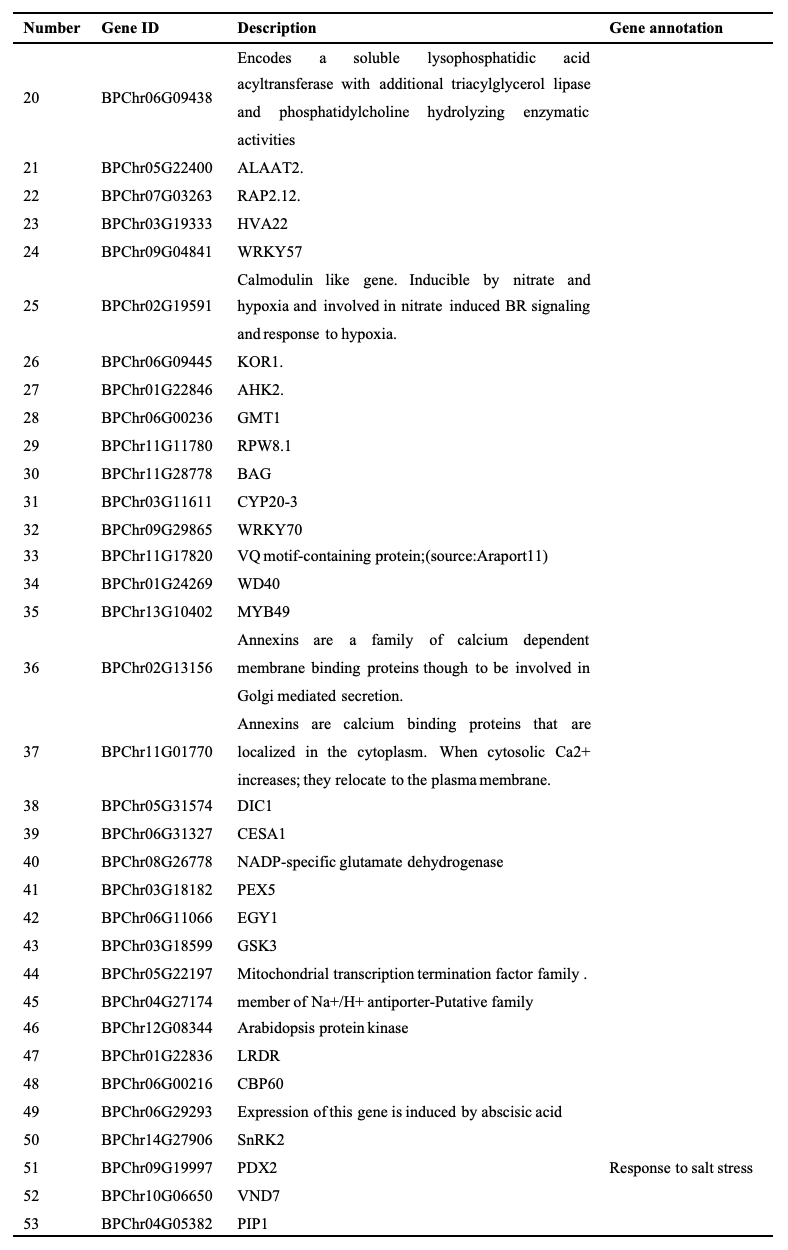

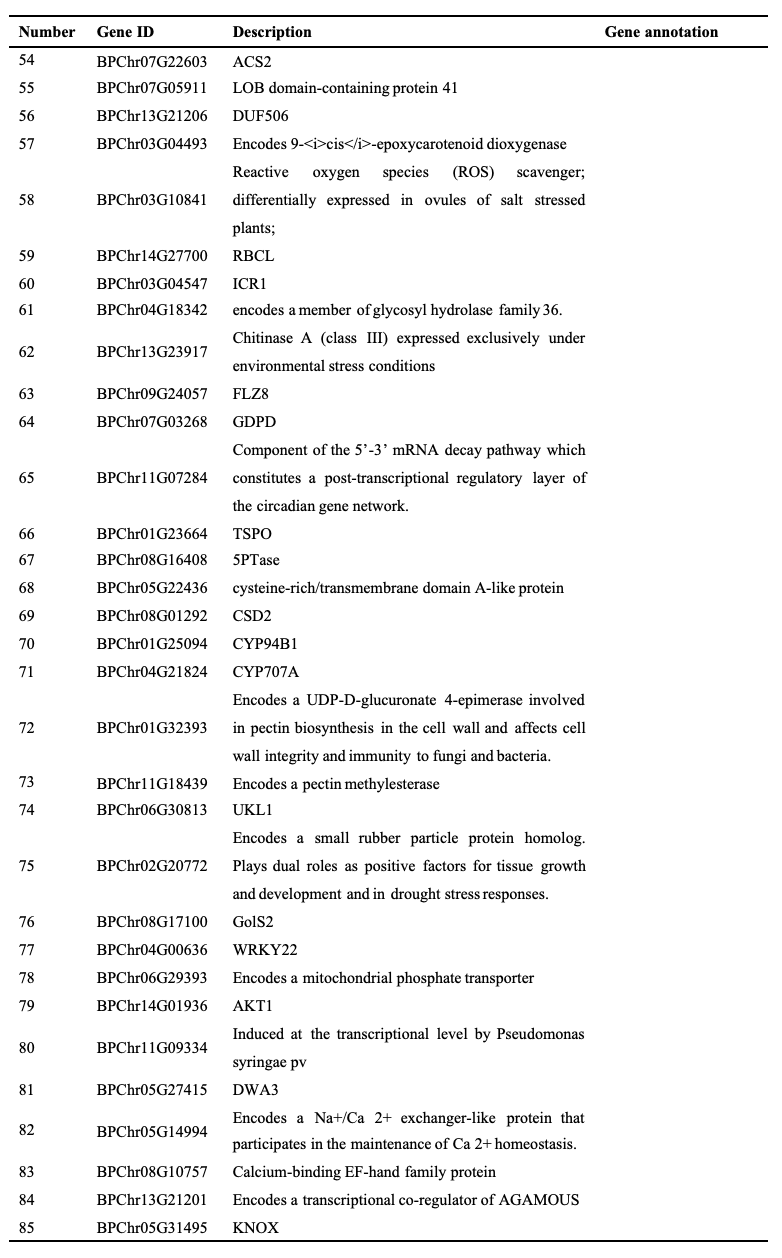

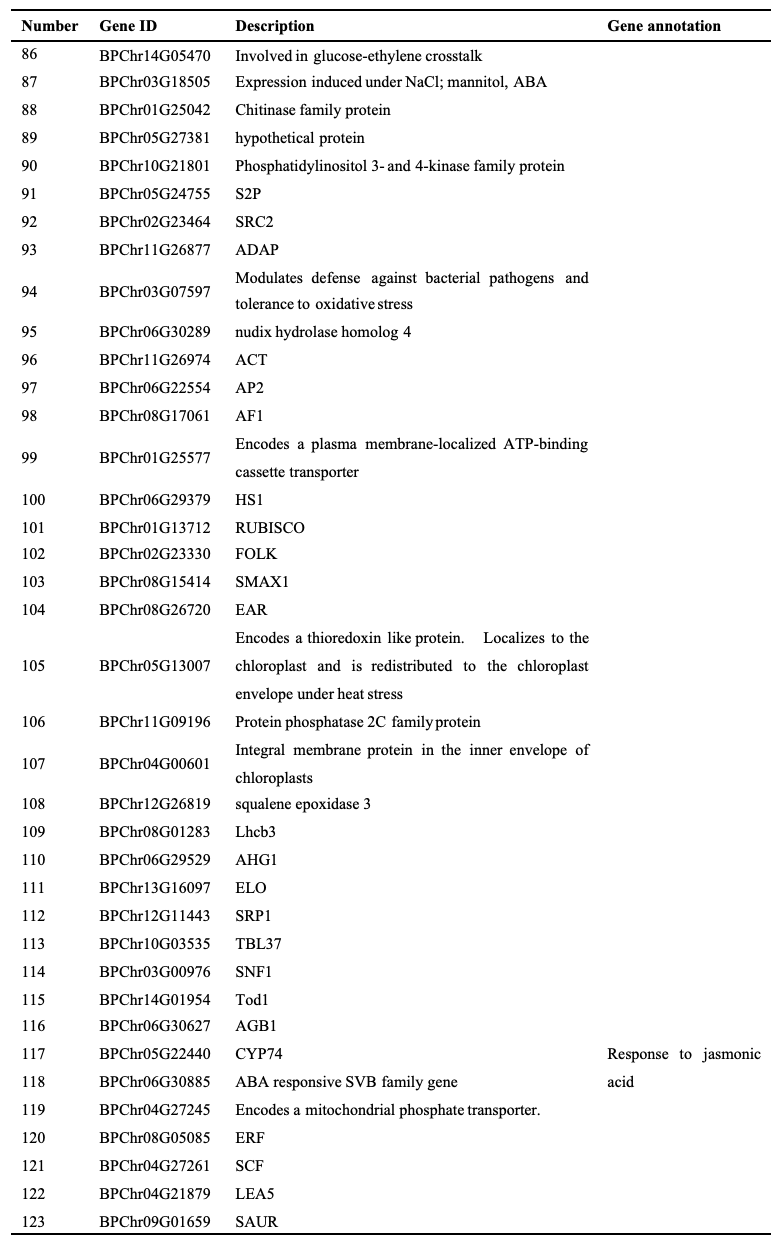

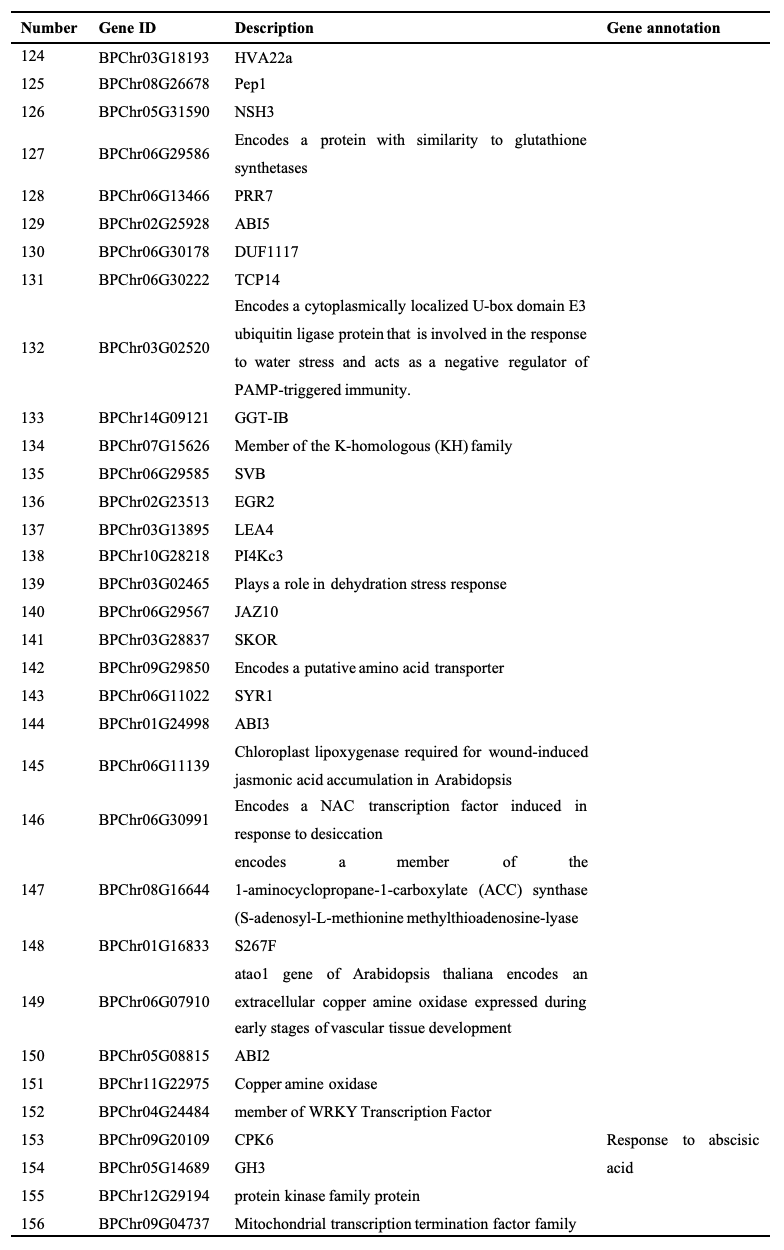

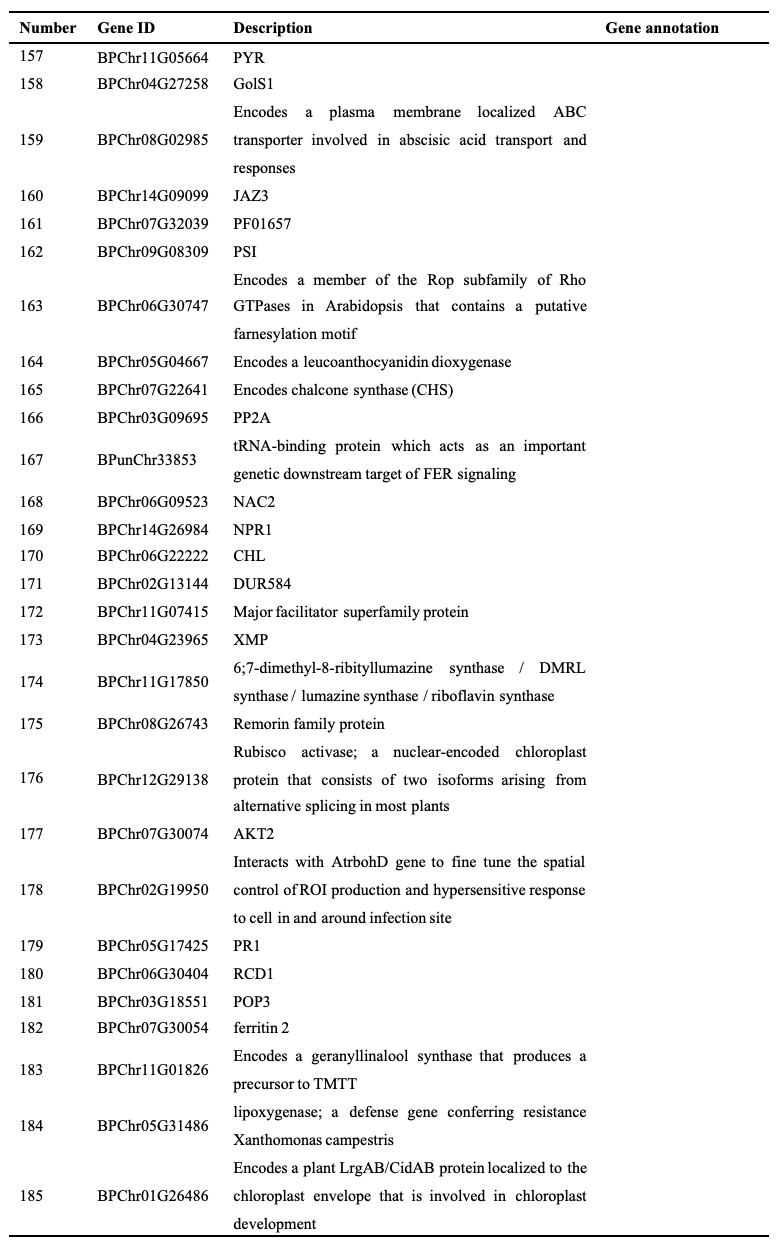

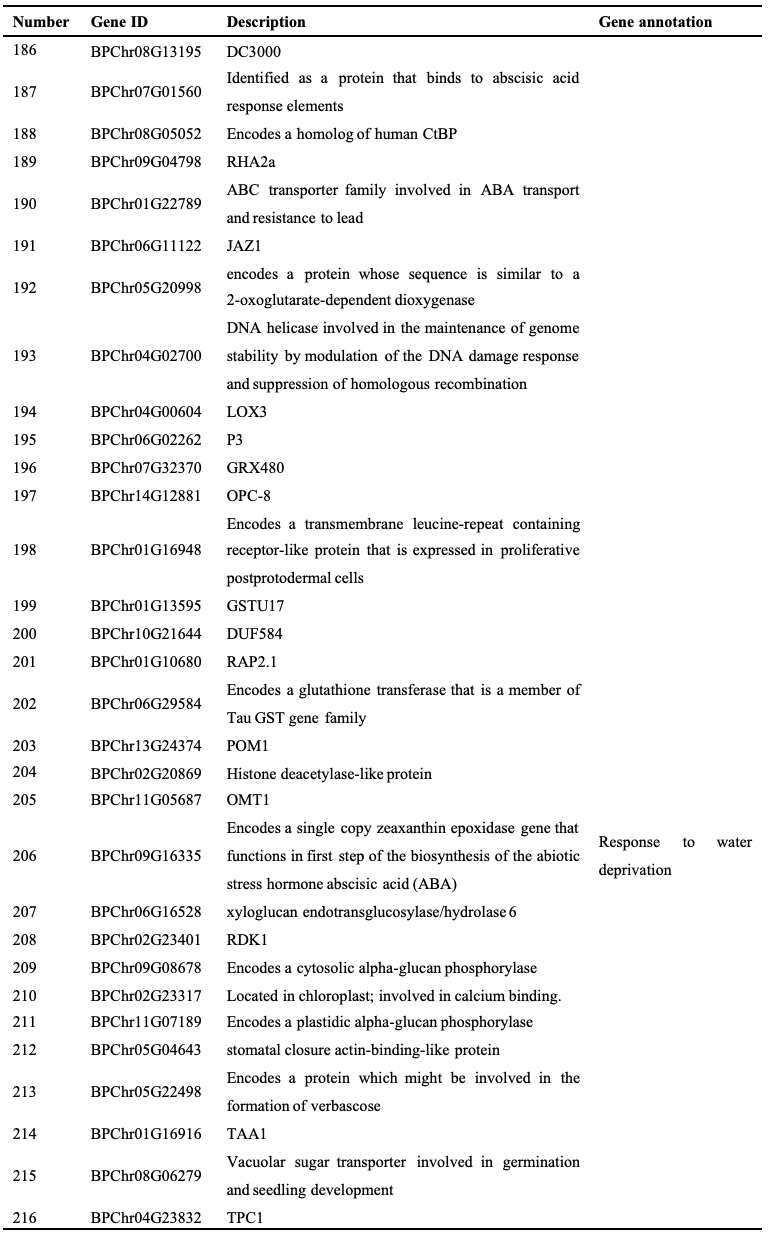

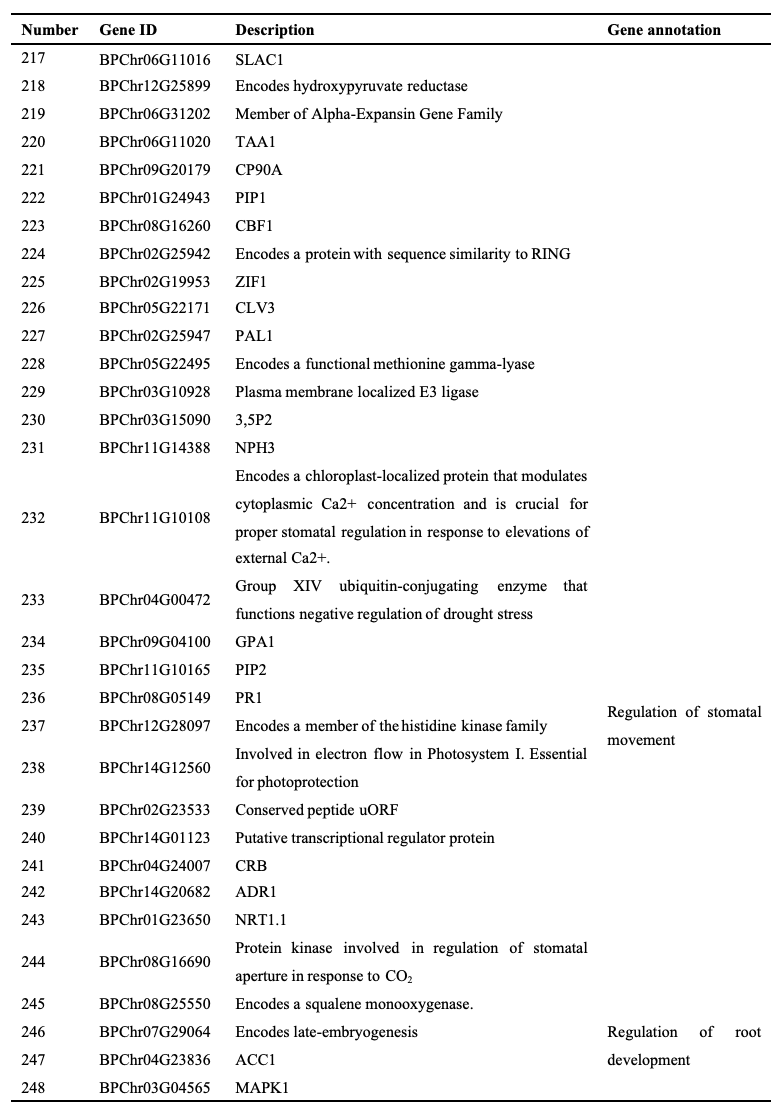

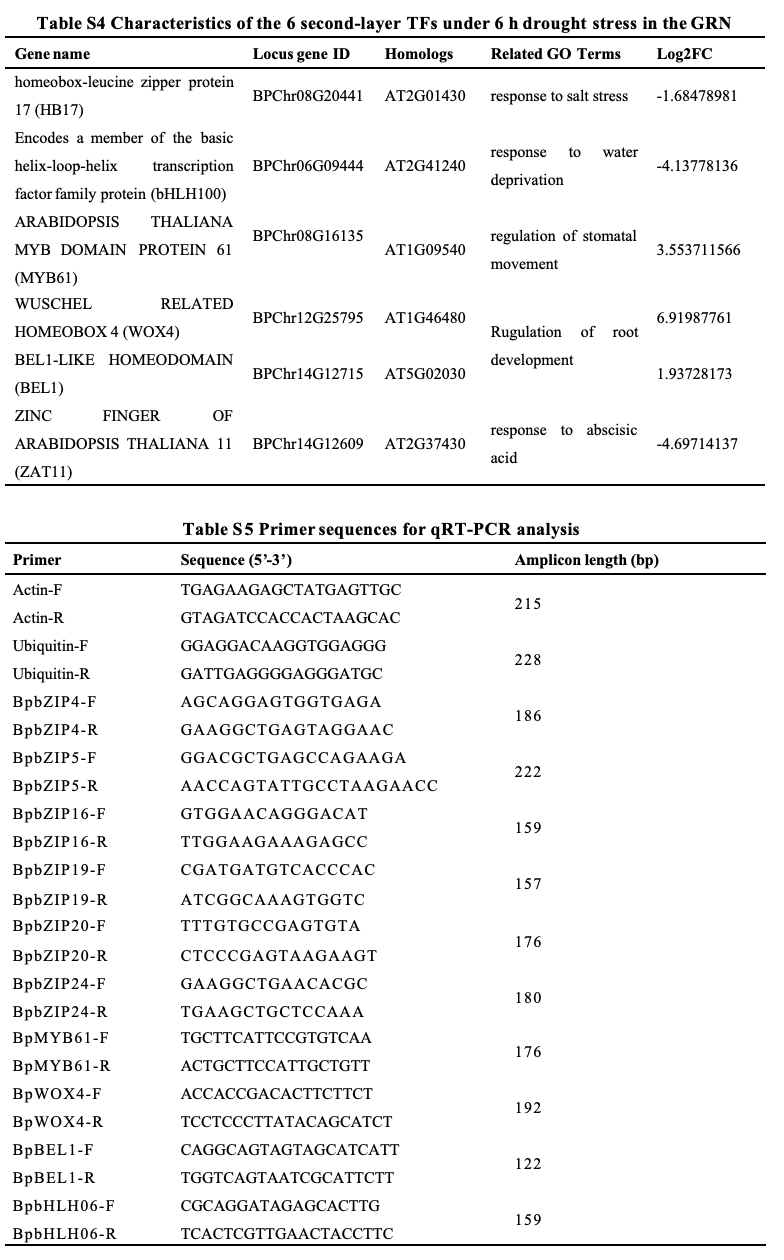

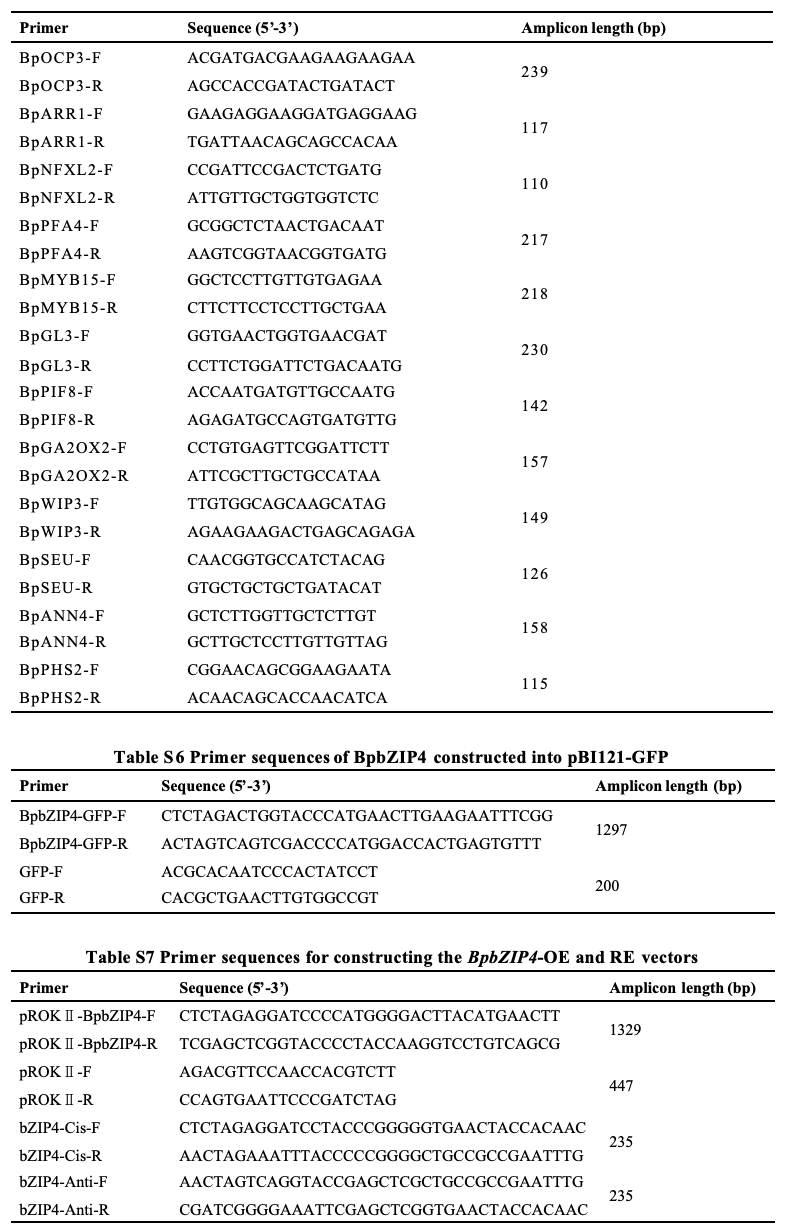

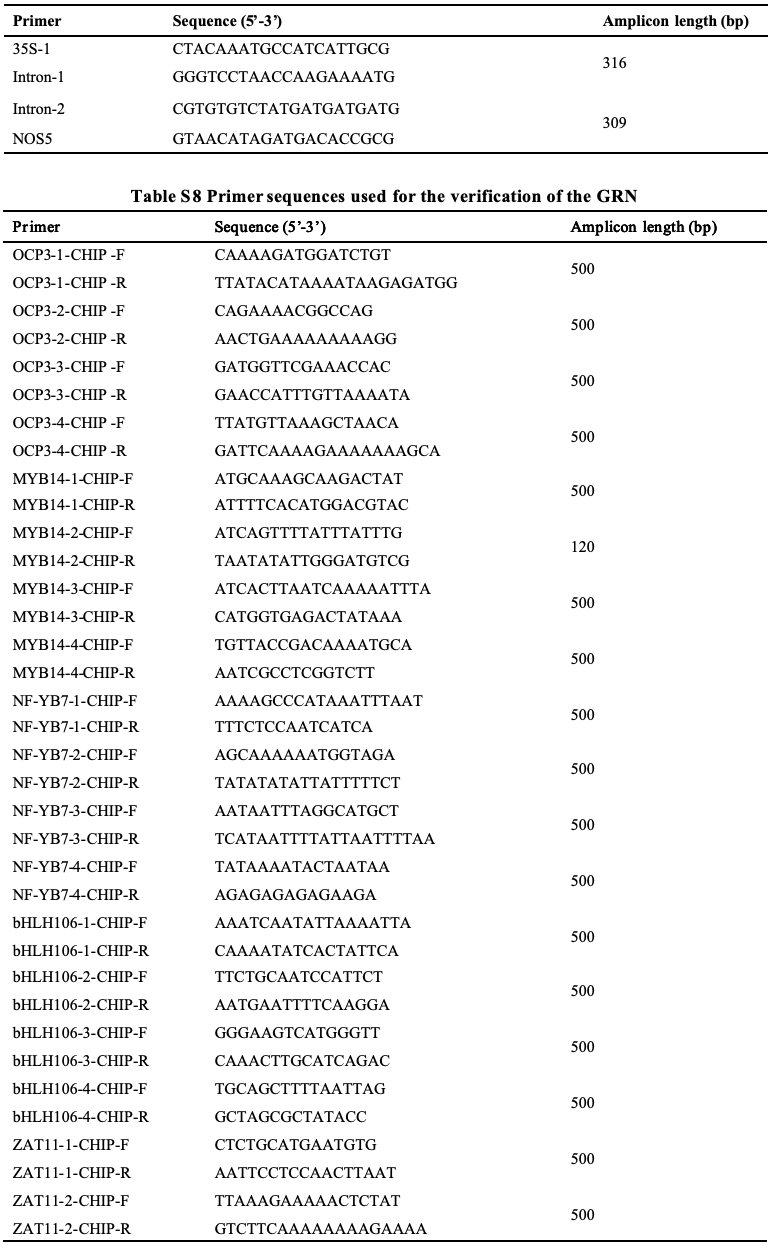

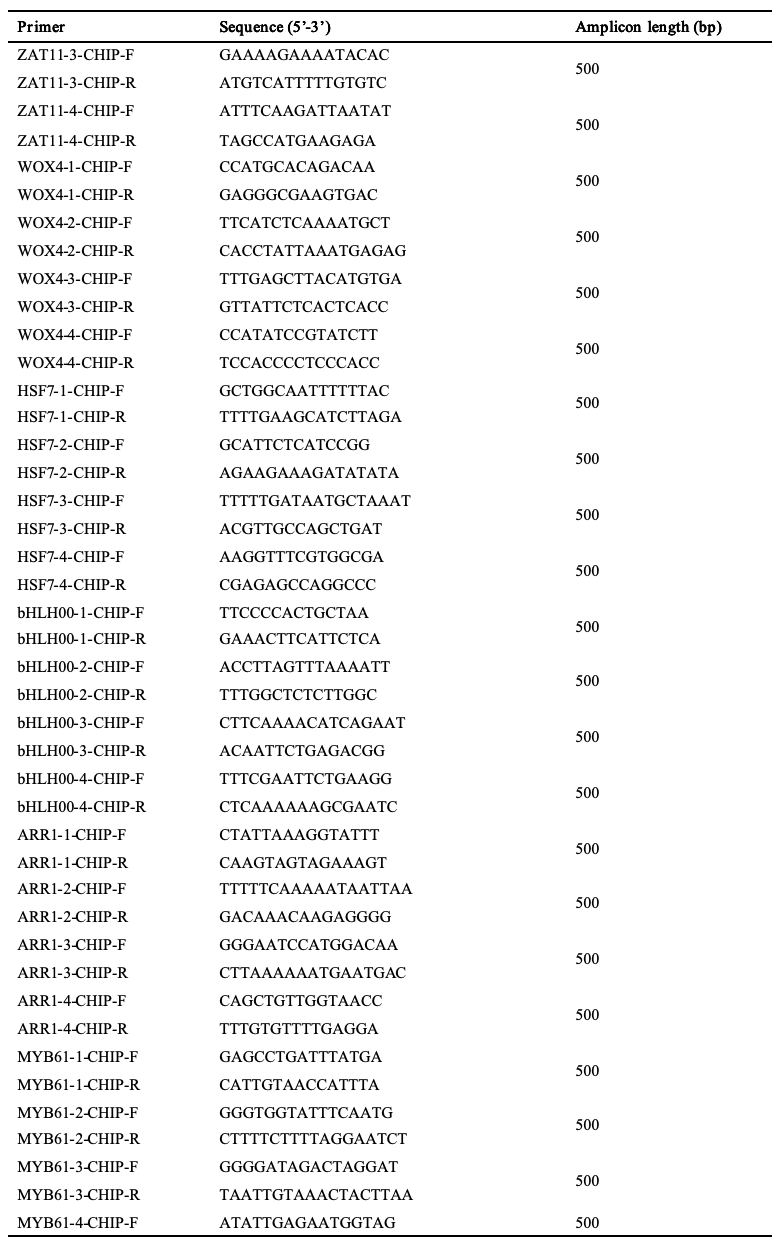

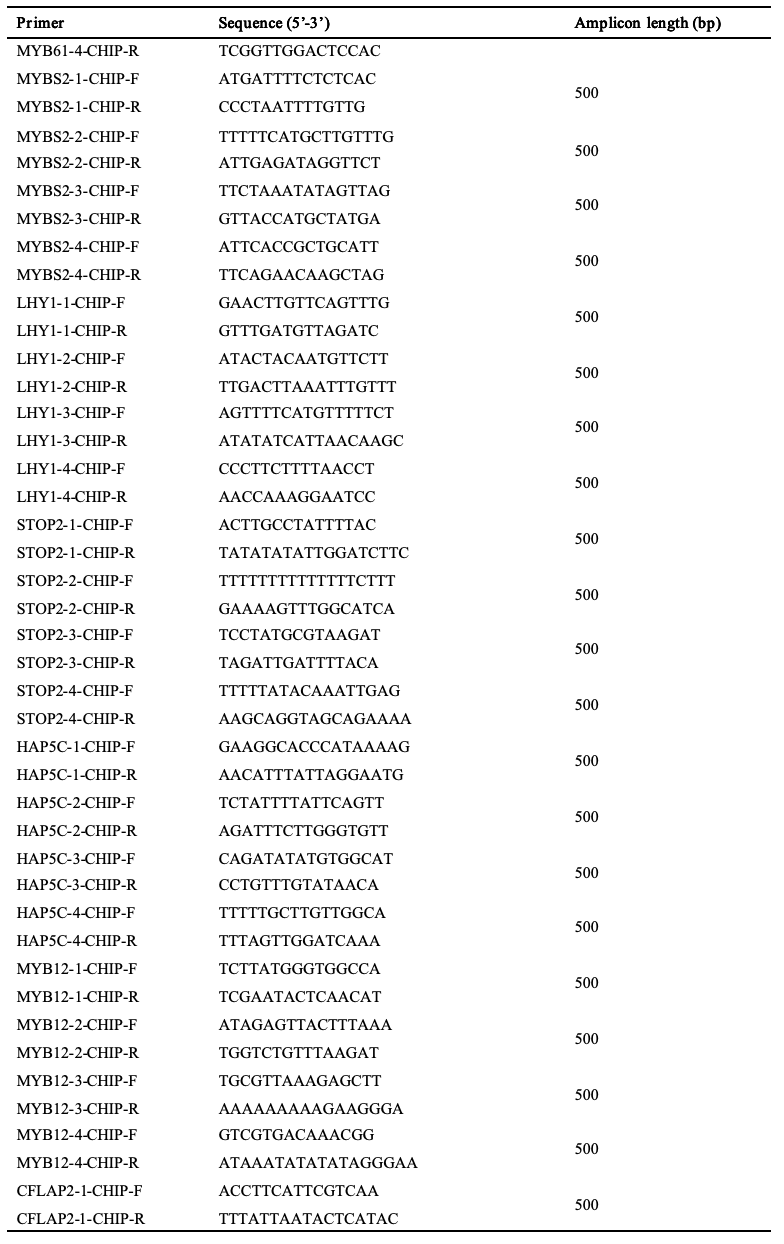

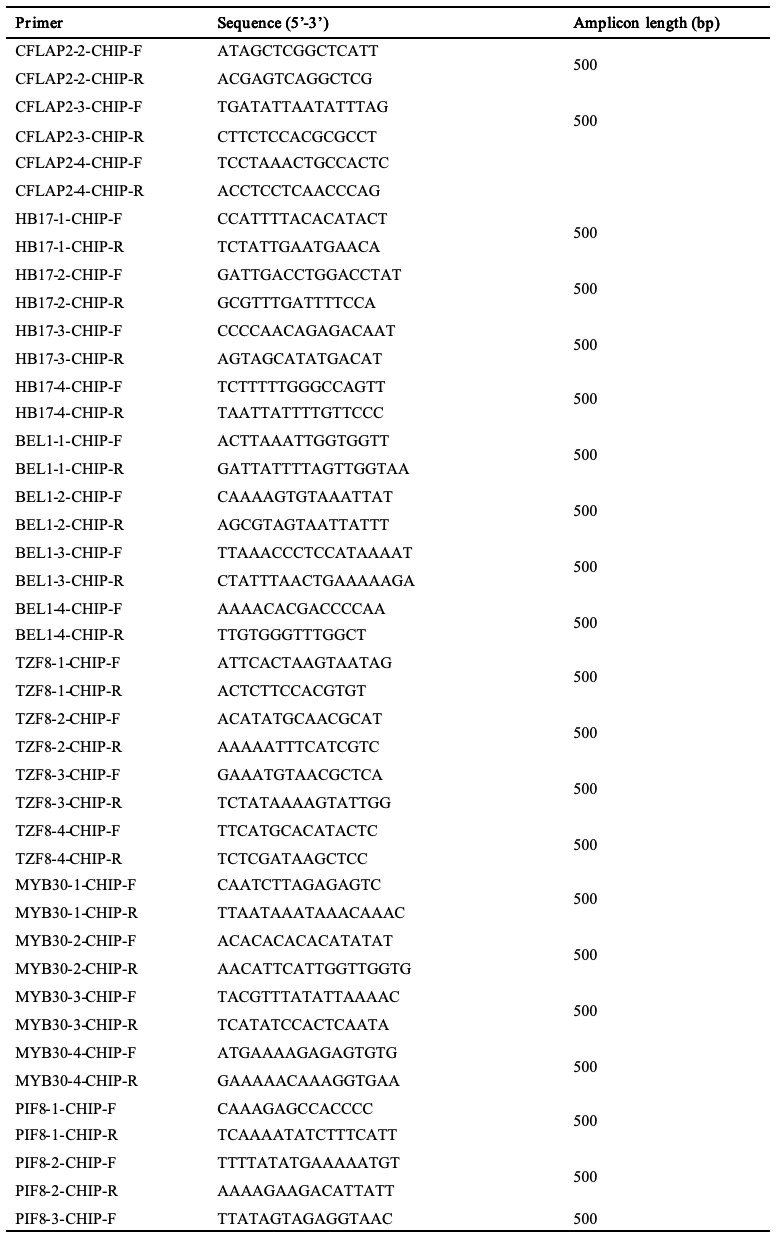

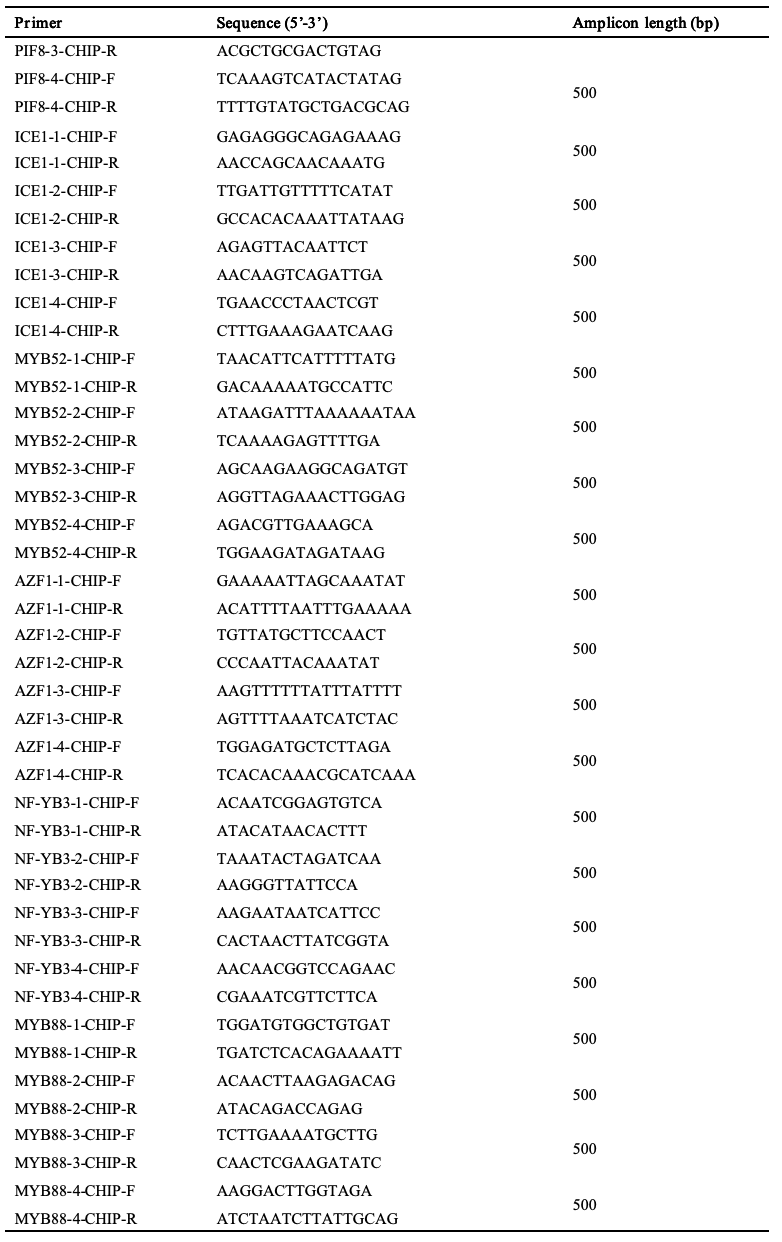

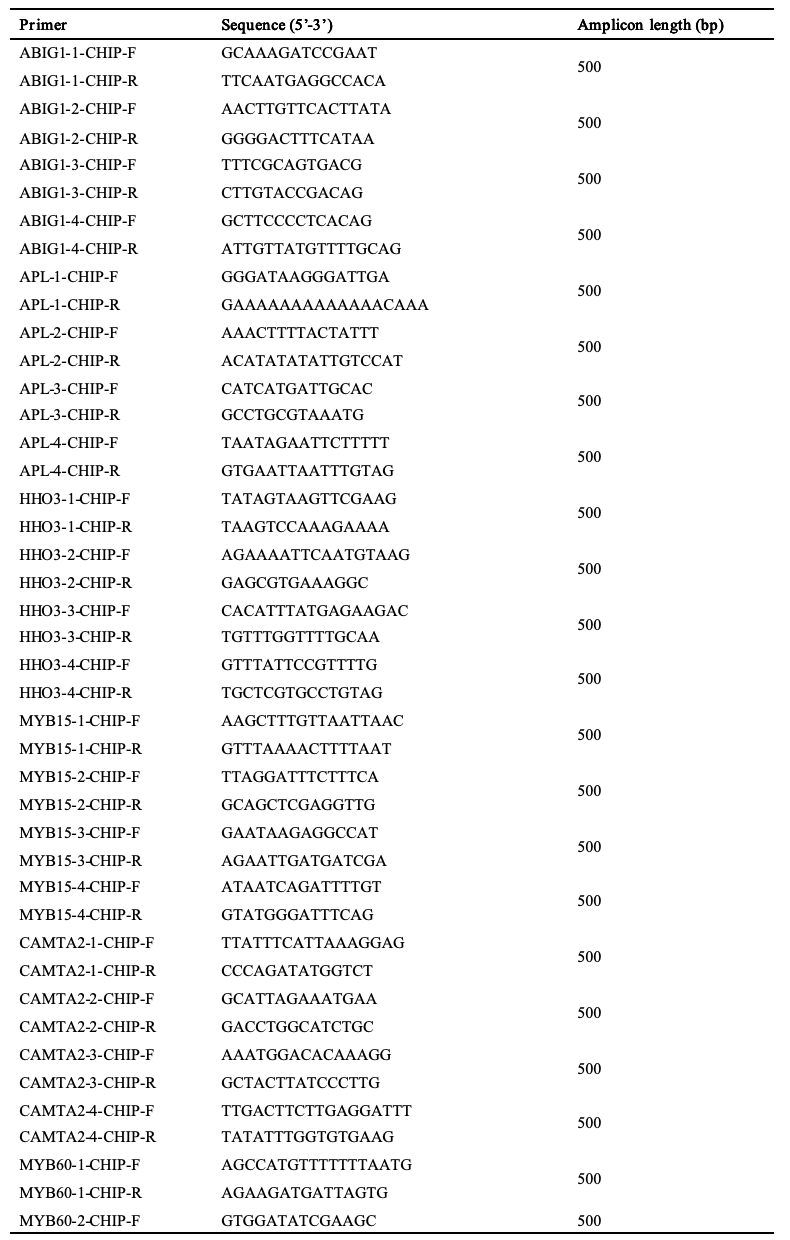

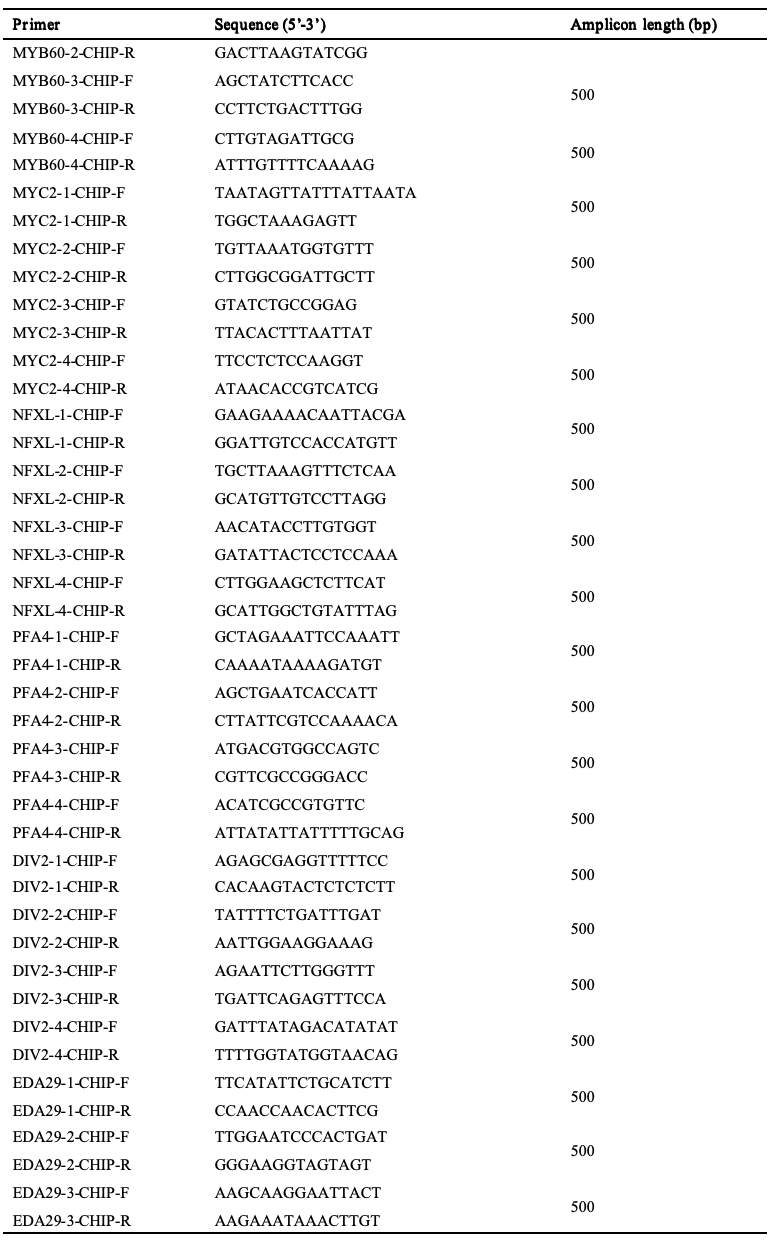

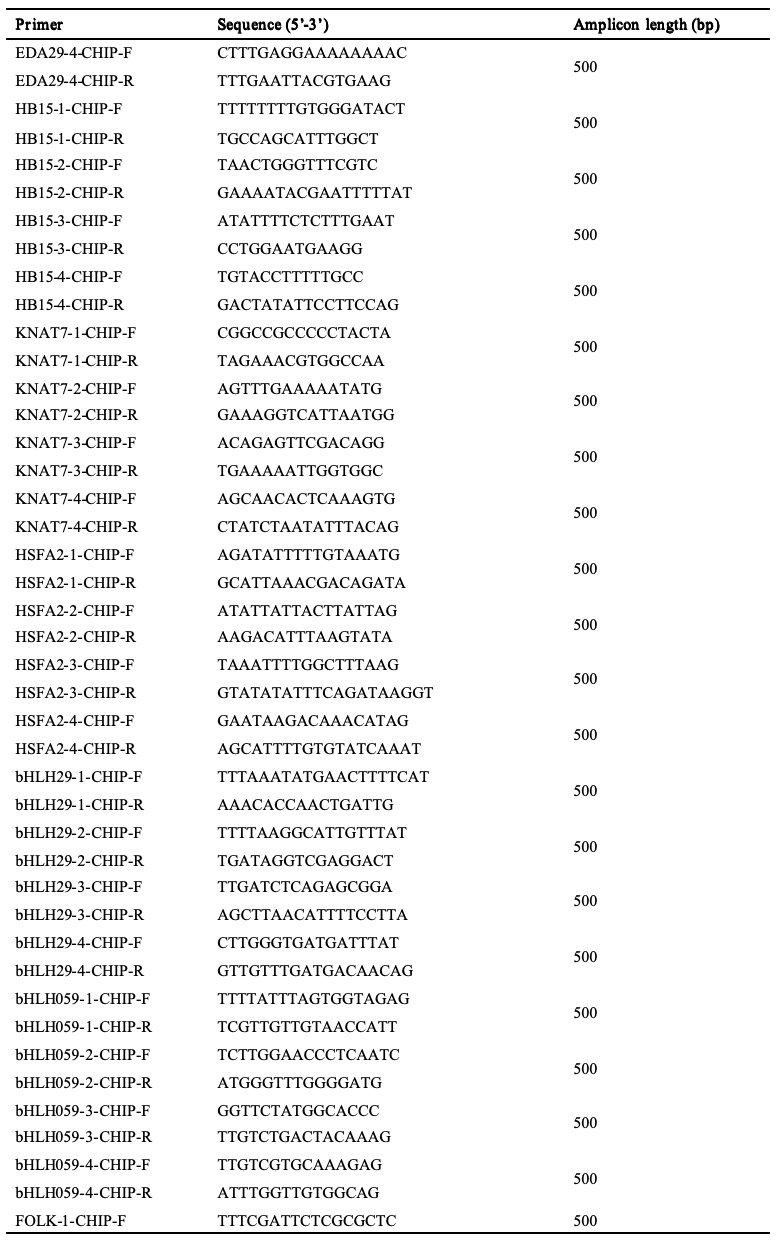

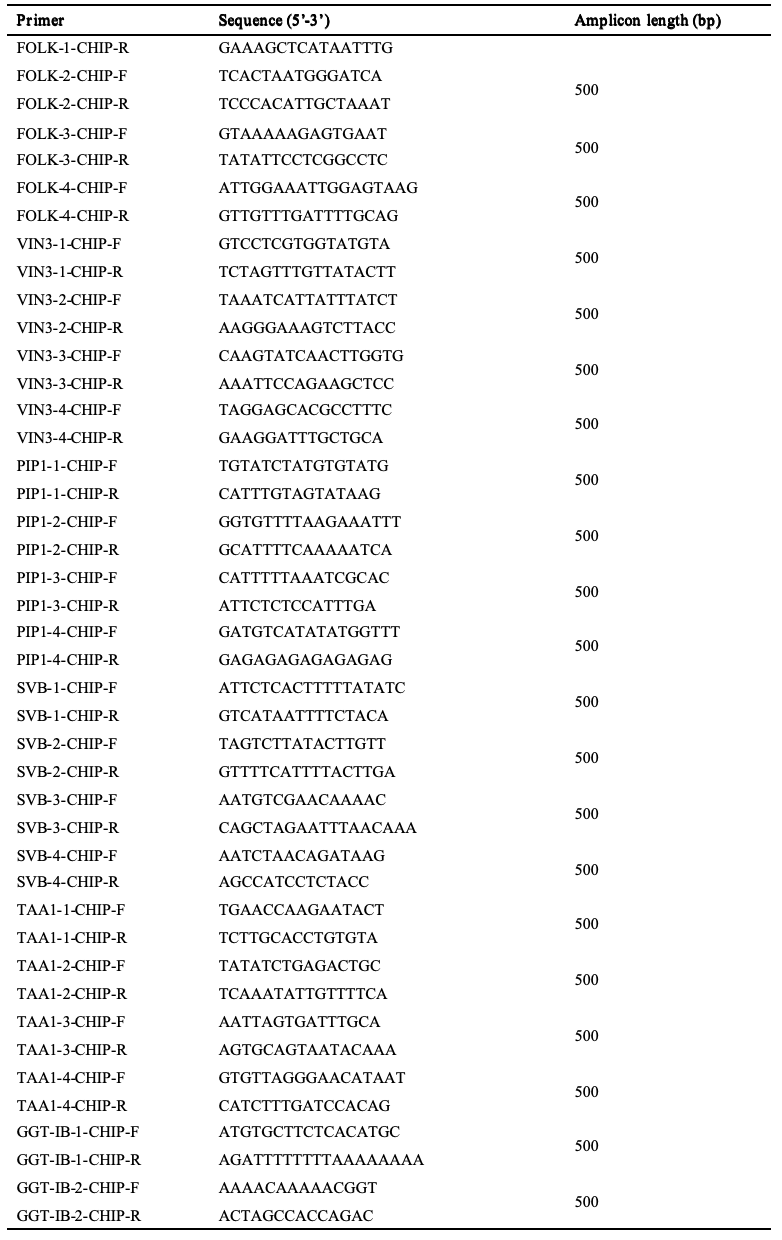

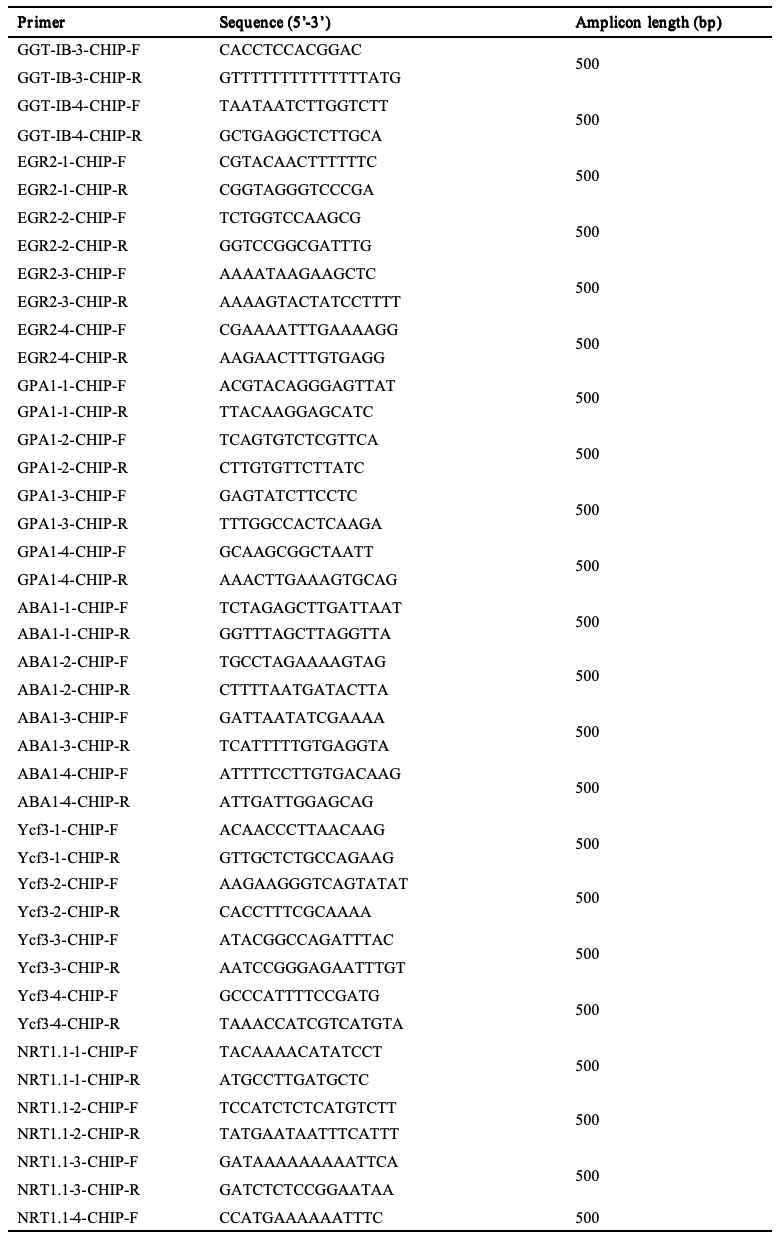

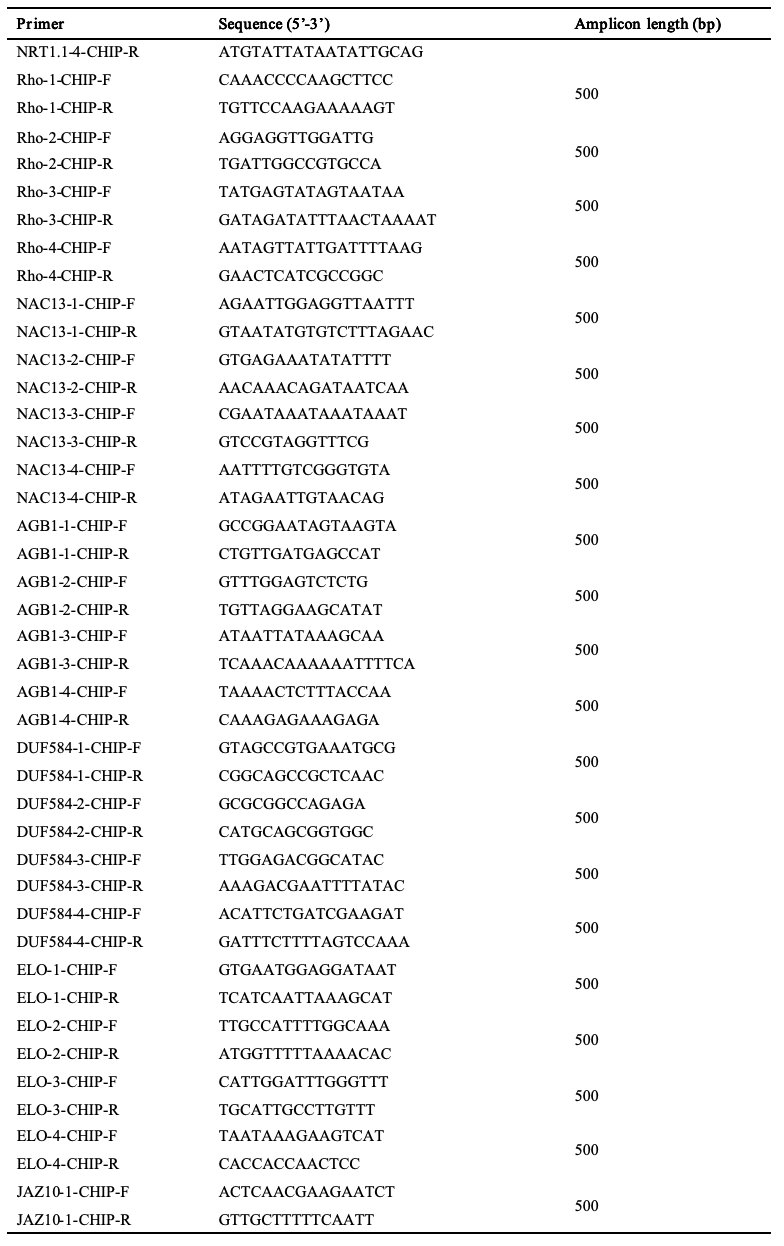

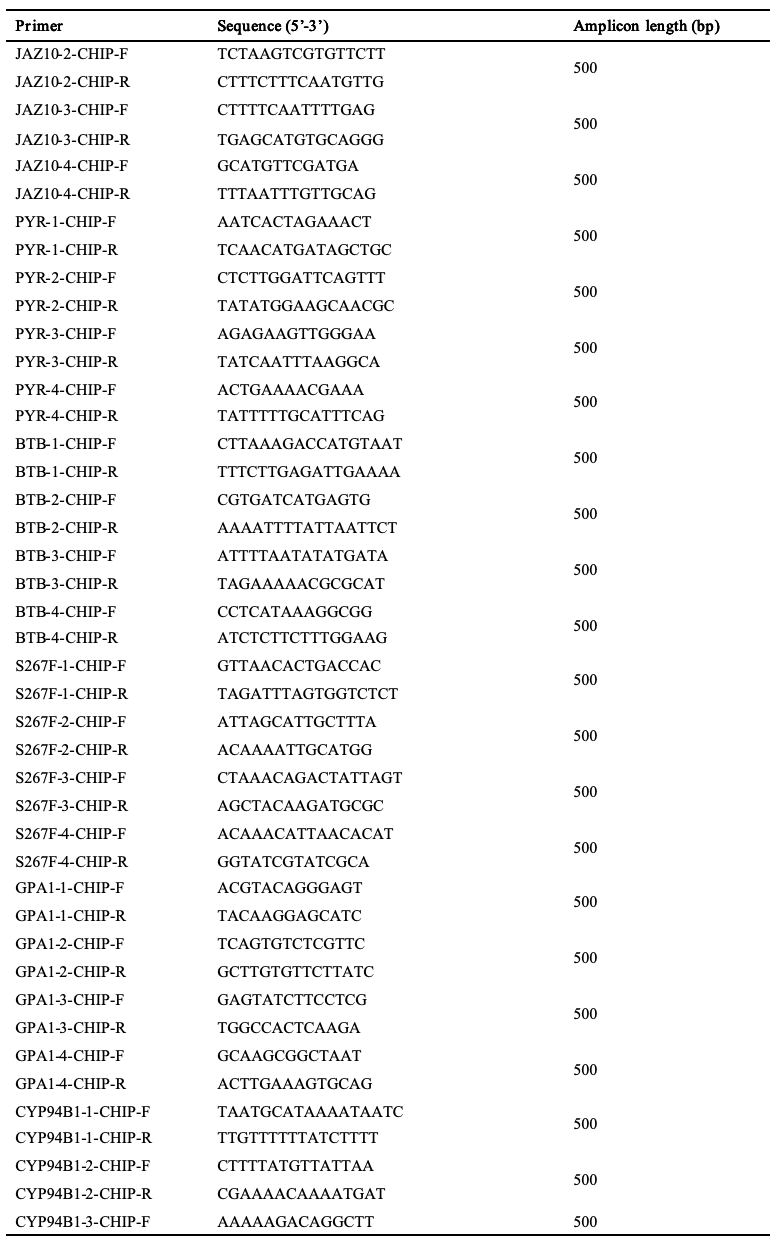


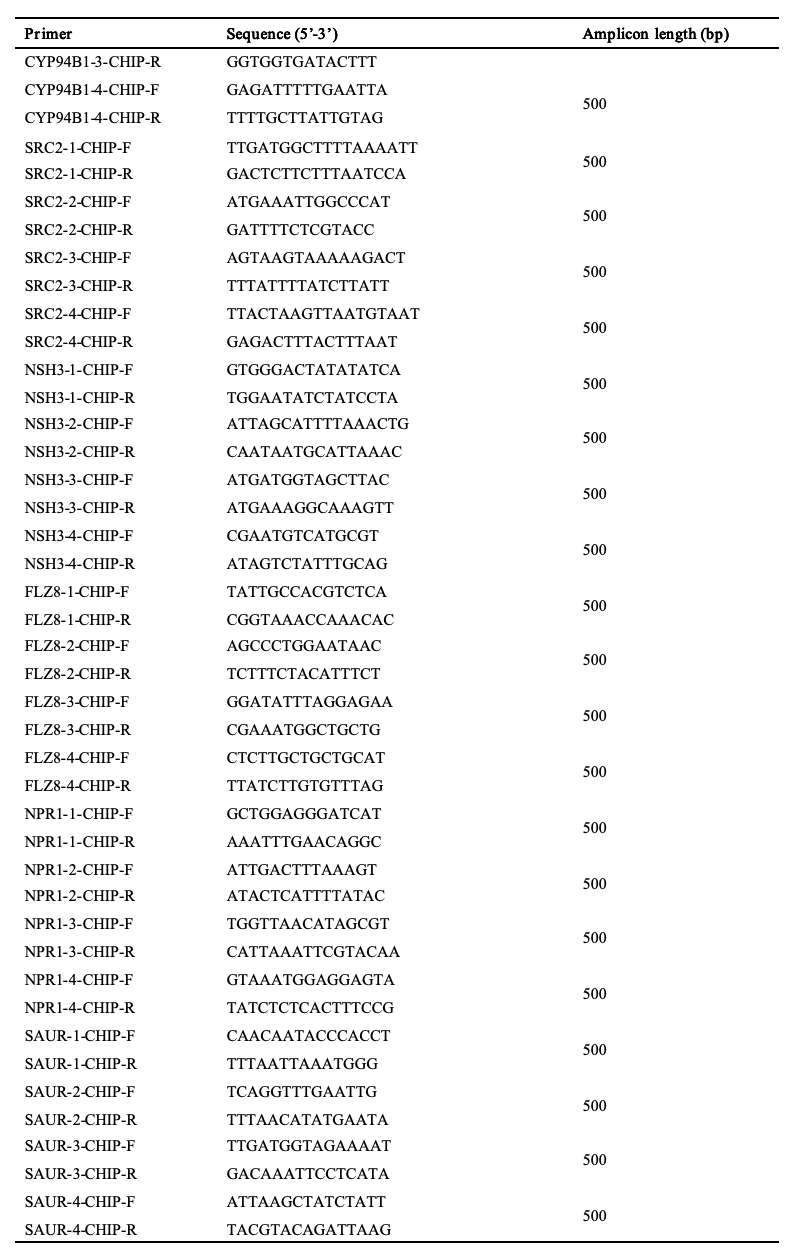

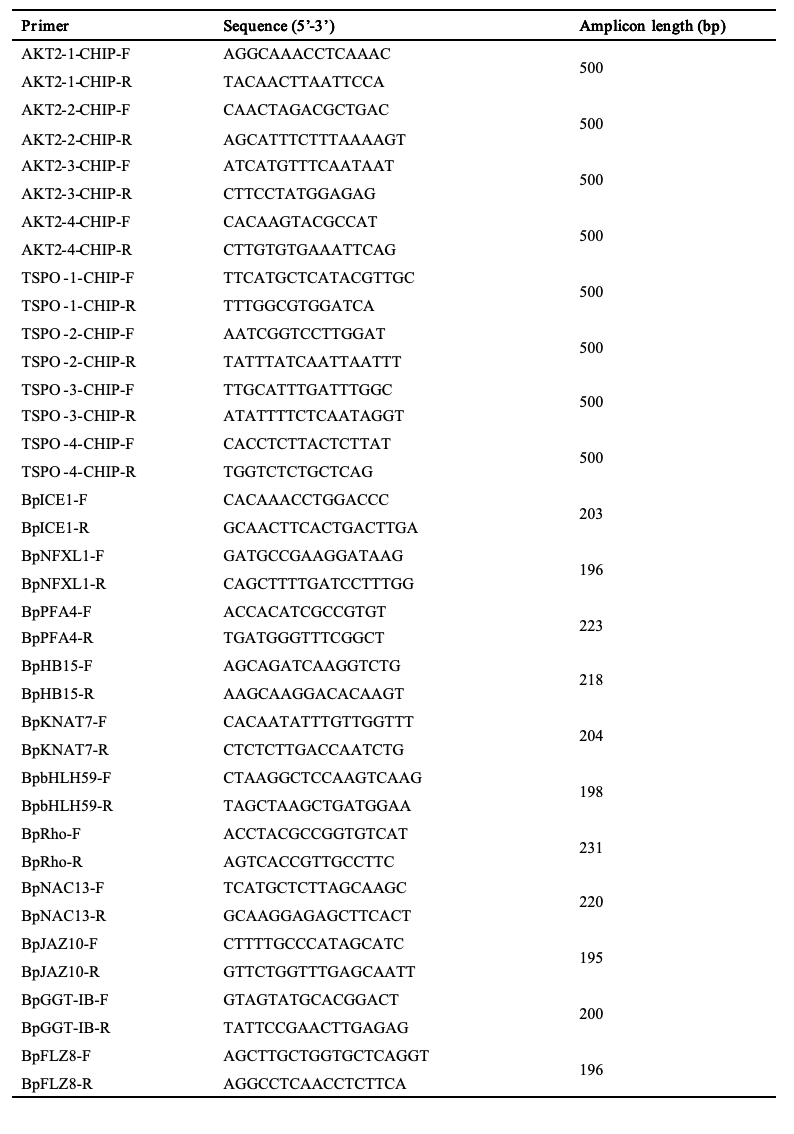

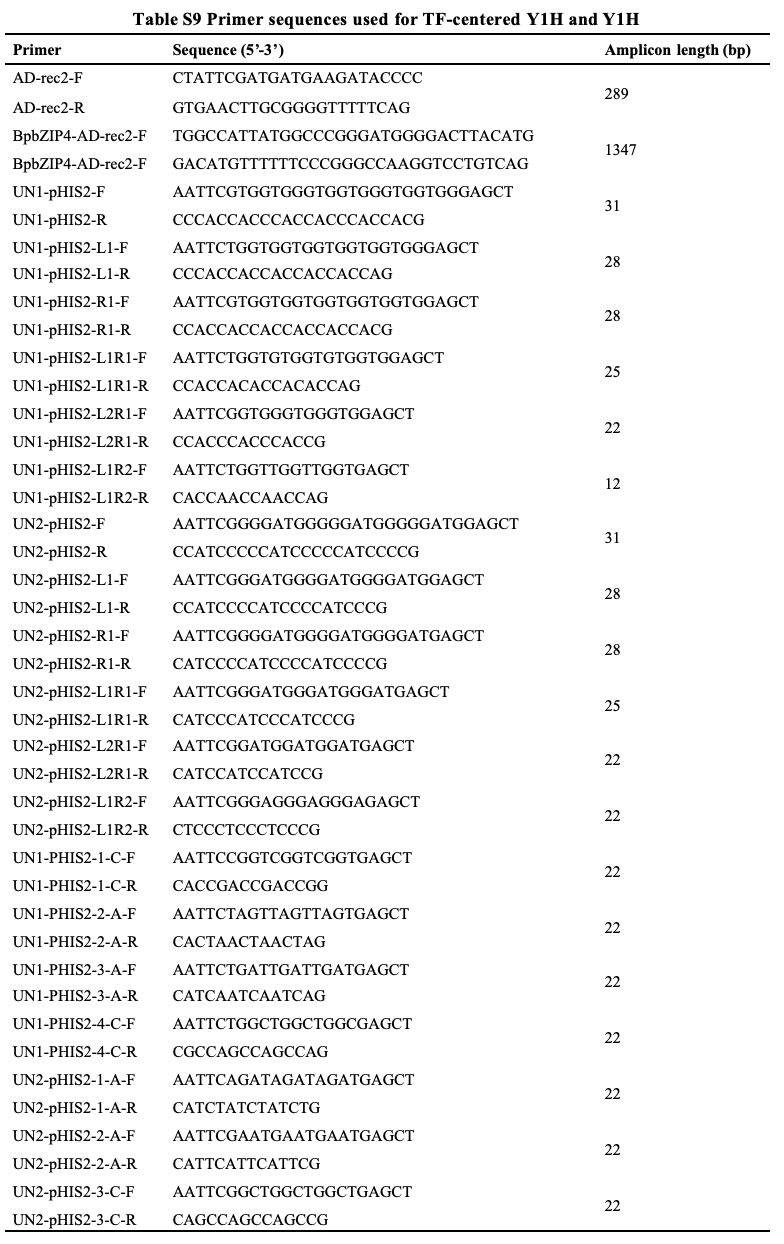

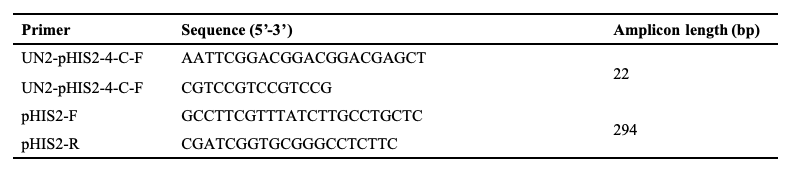

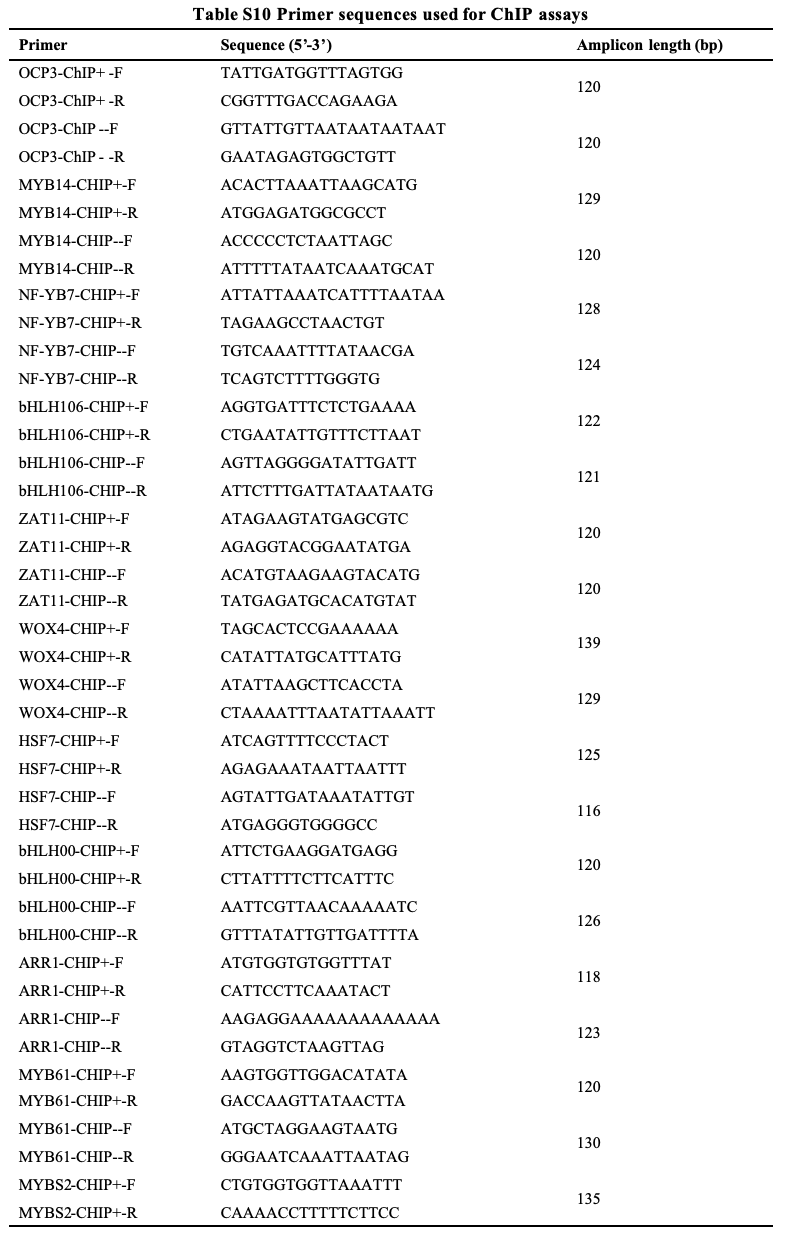

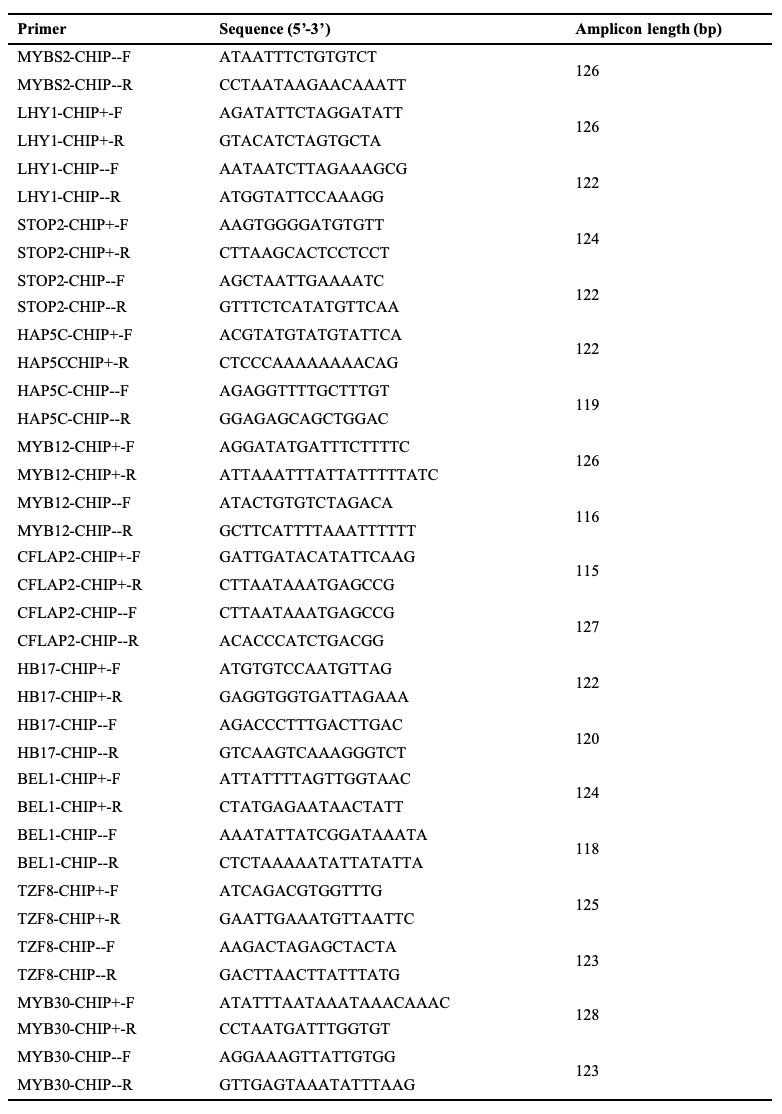

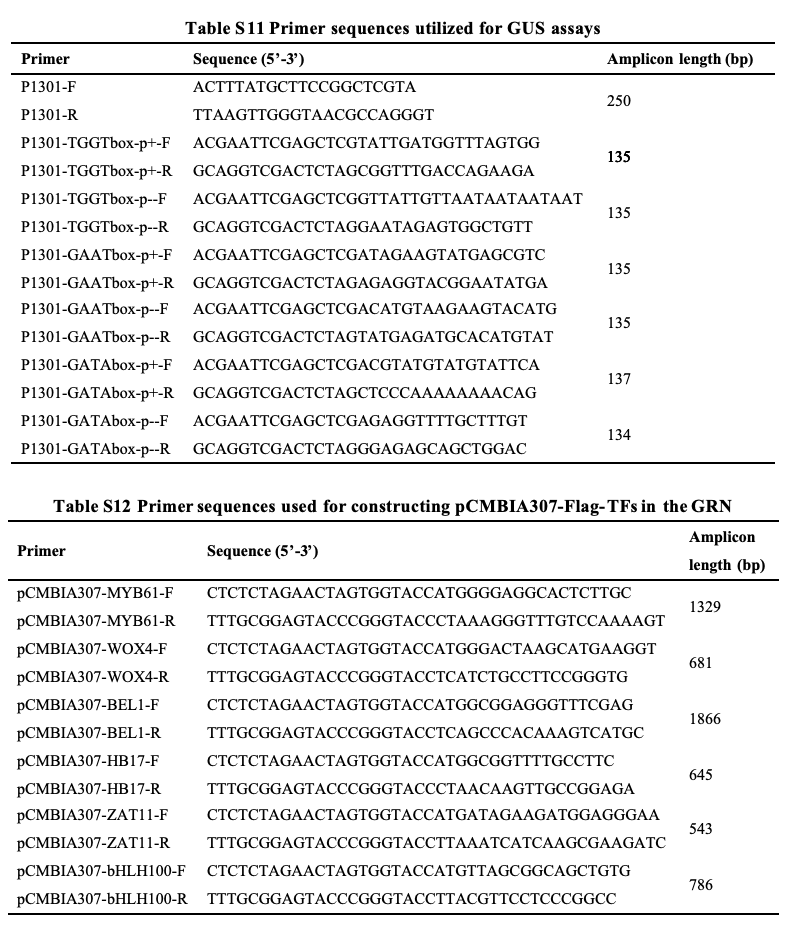

Supplement: Web_Material_uhag002 [file web_material_uhag002.zip › BpbZIP4-Table-HR-12.10.docx]

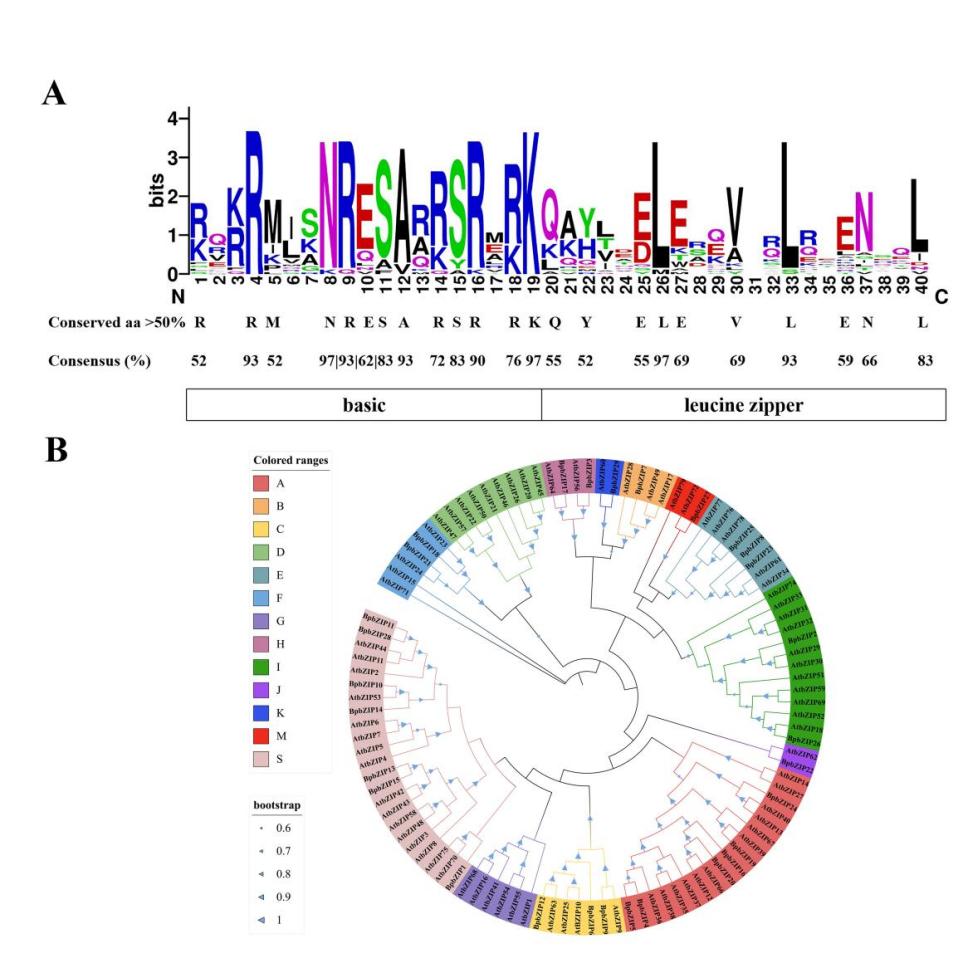


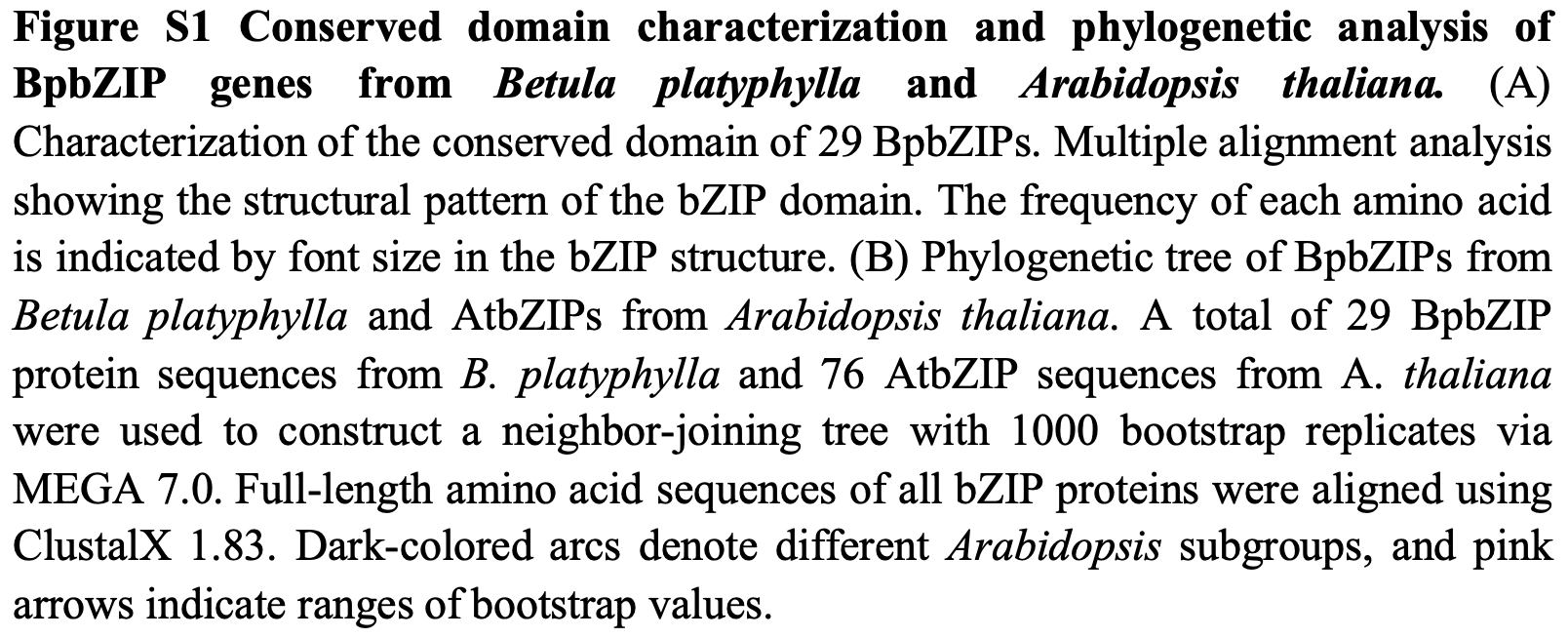


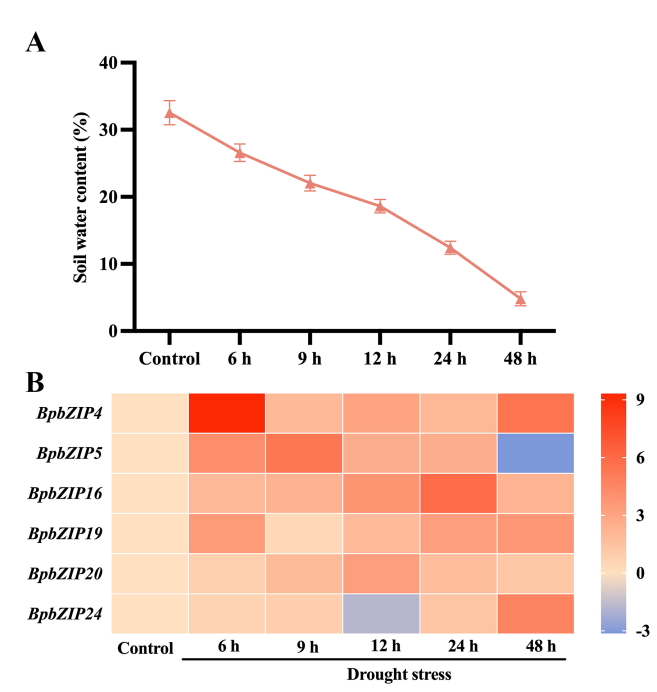


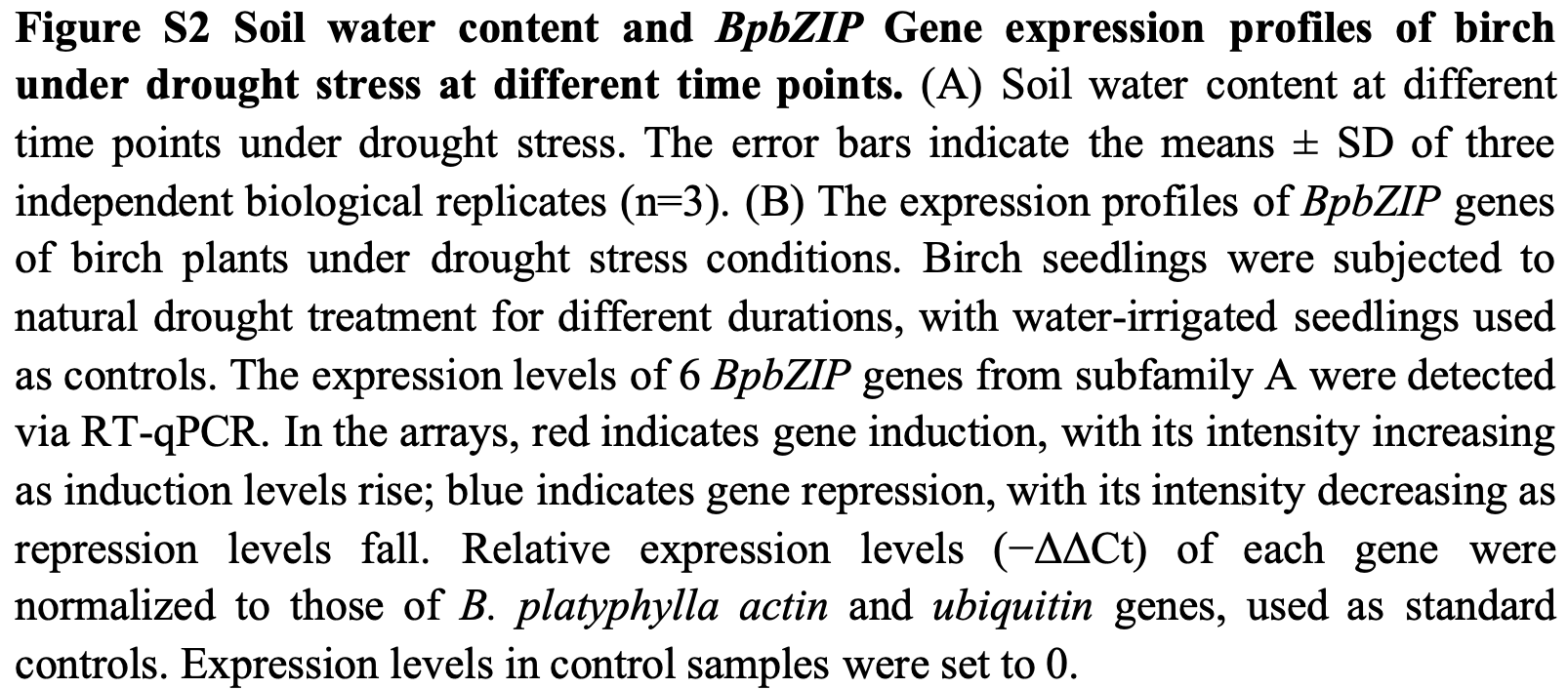


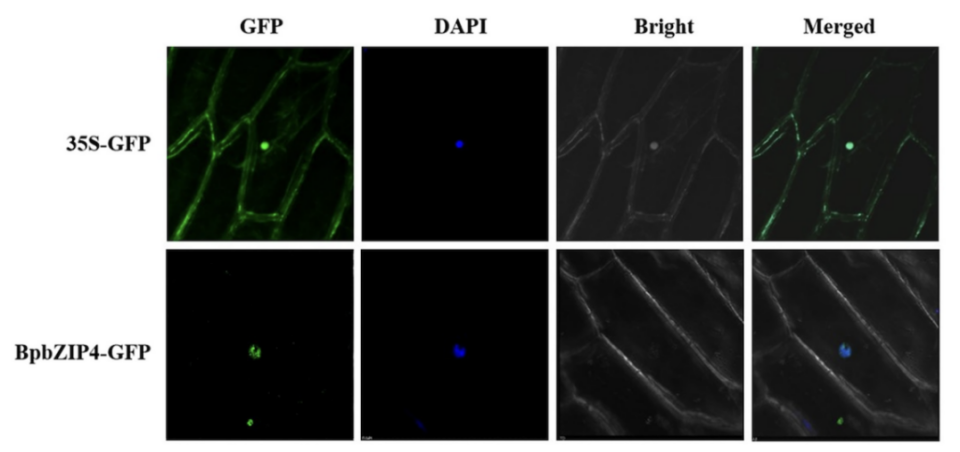


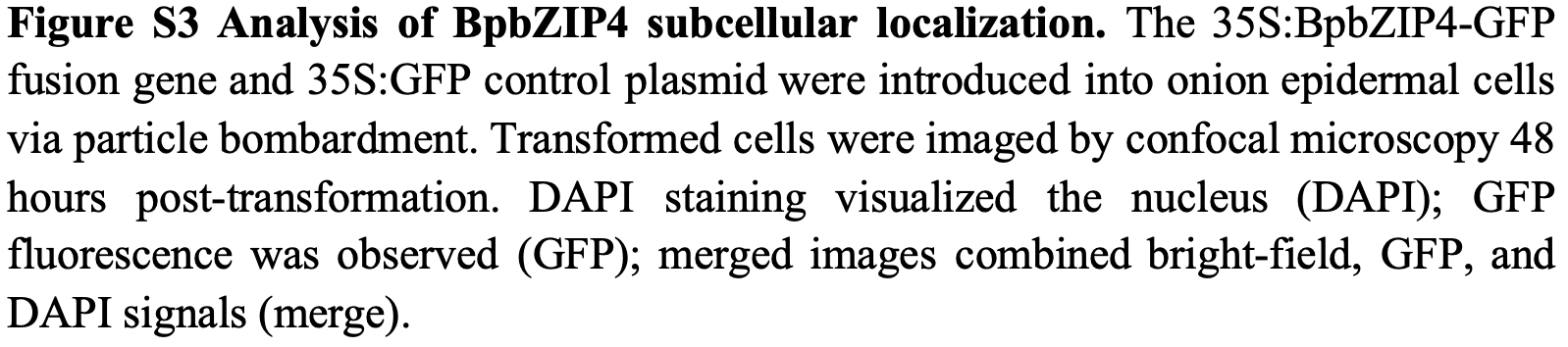


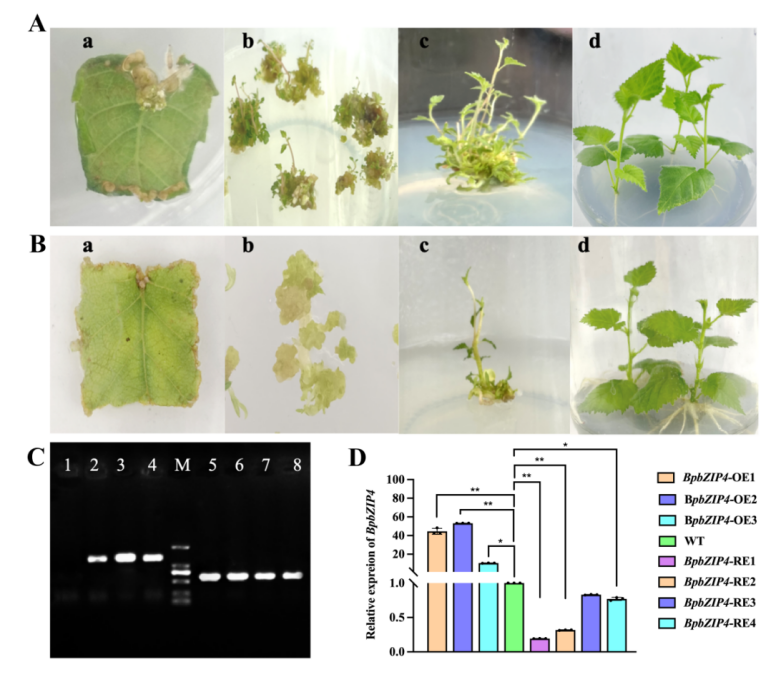


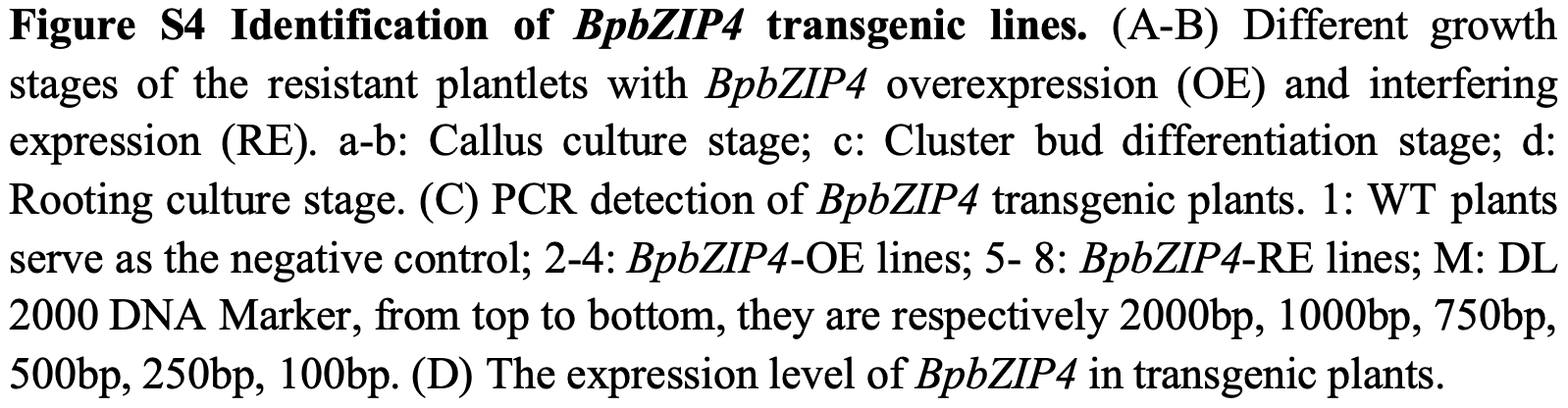


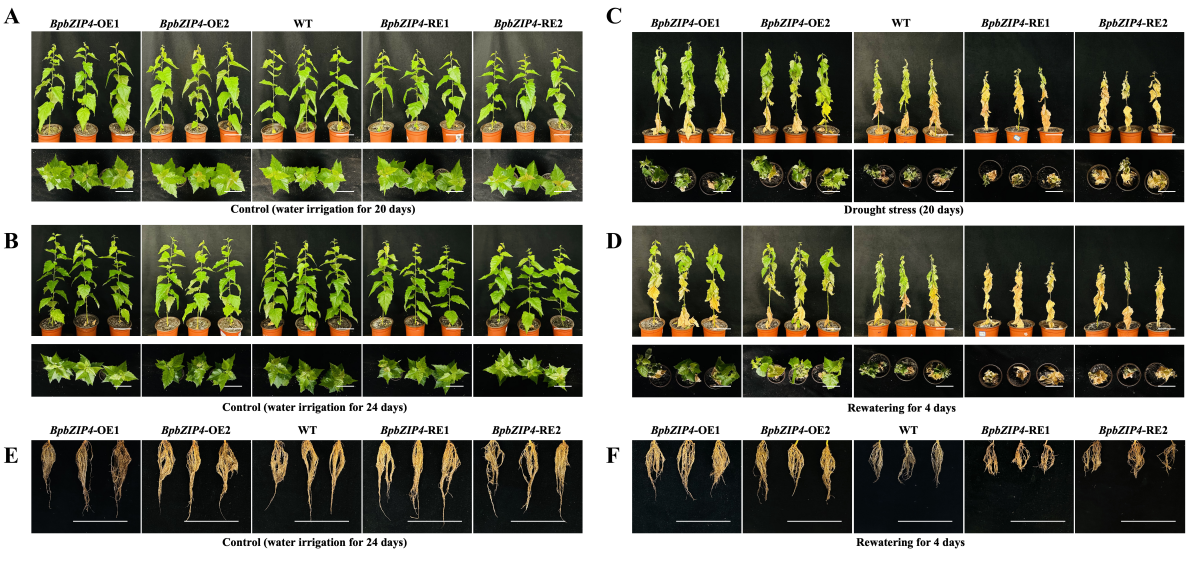


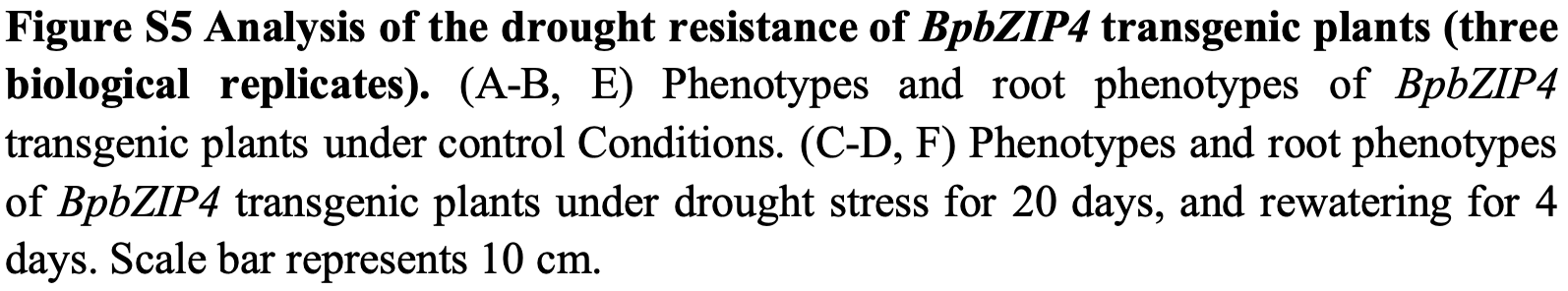


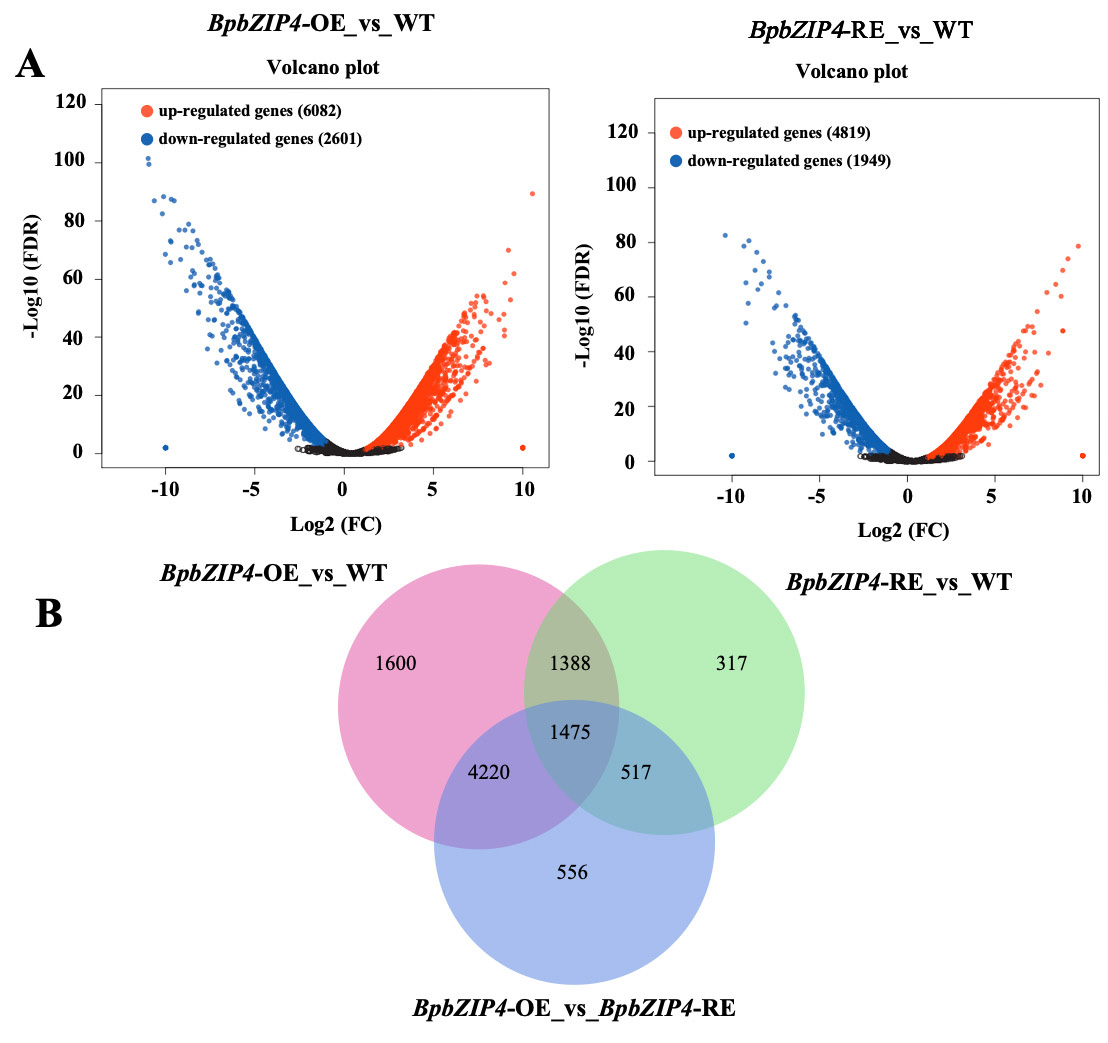


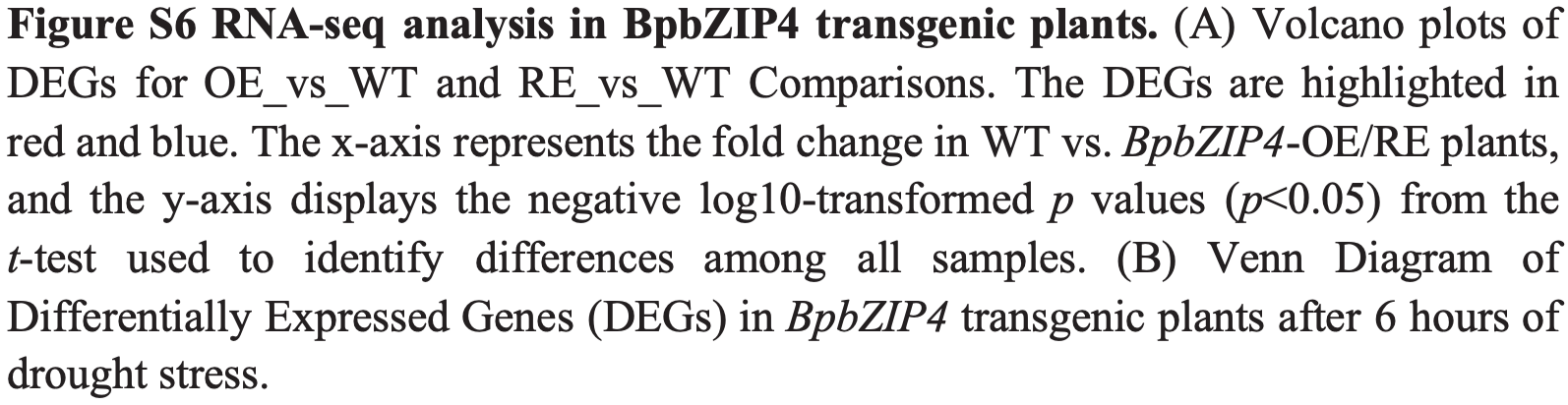


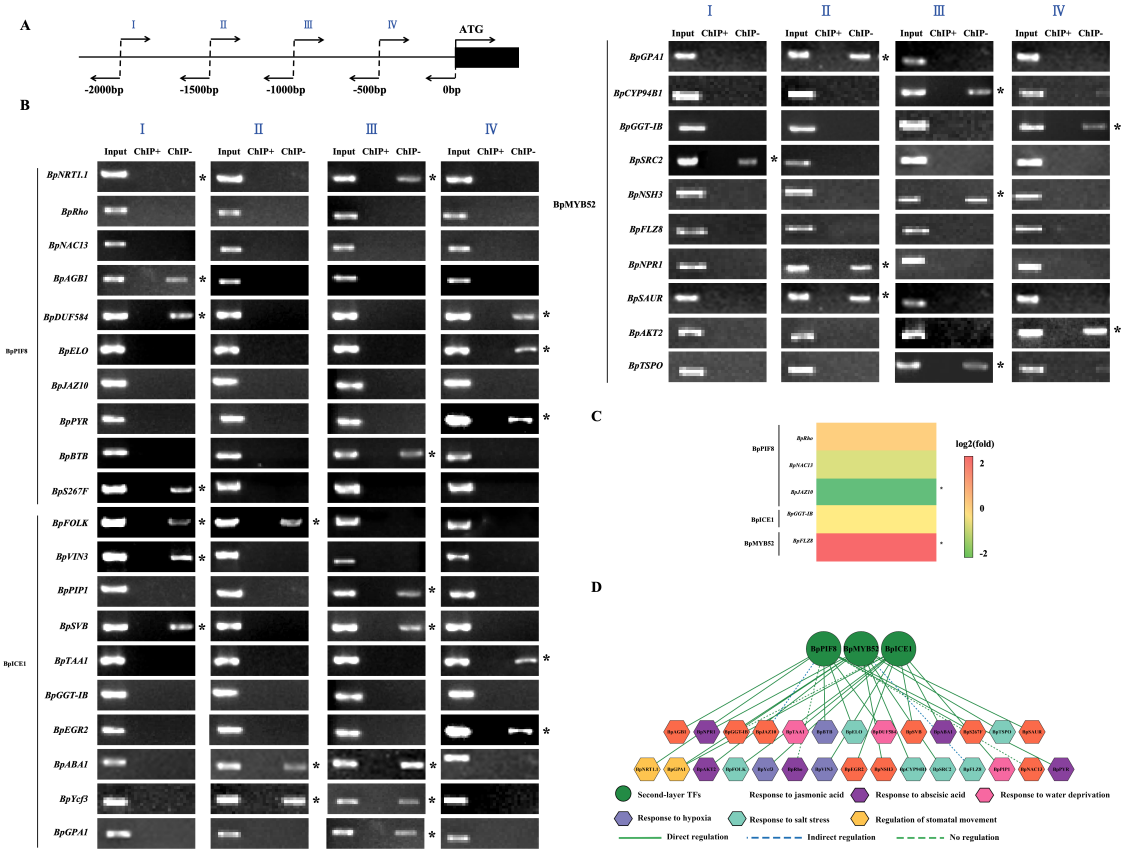


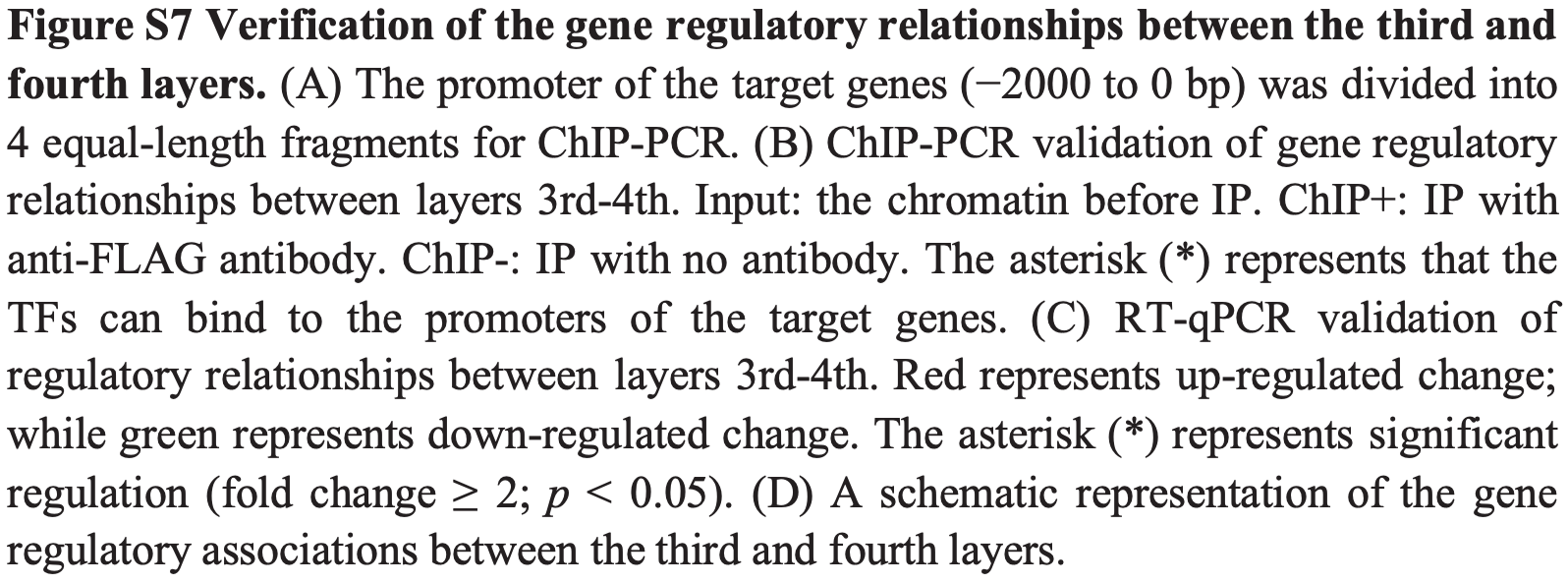


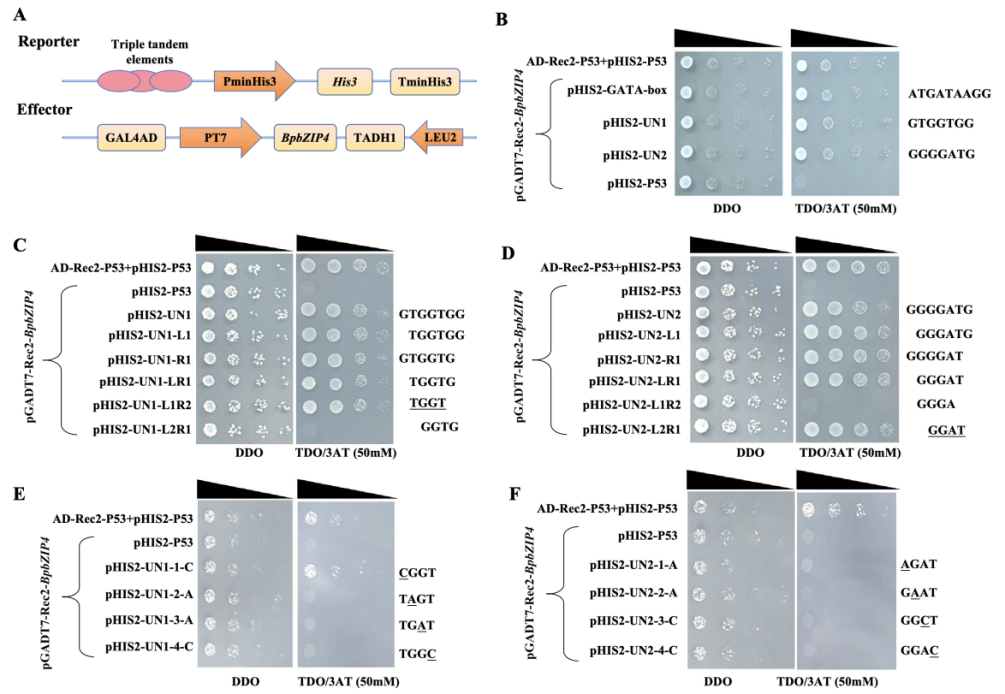


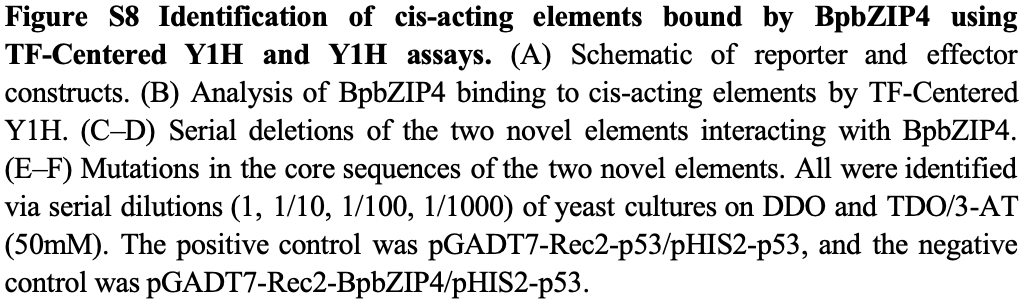


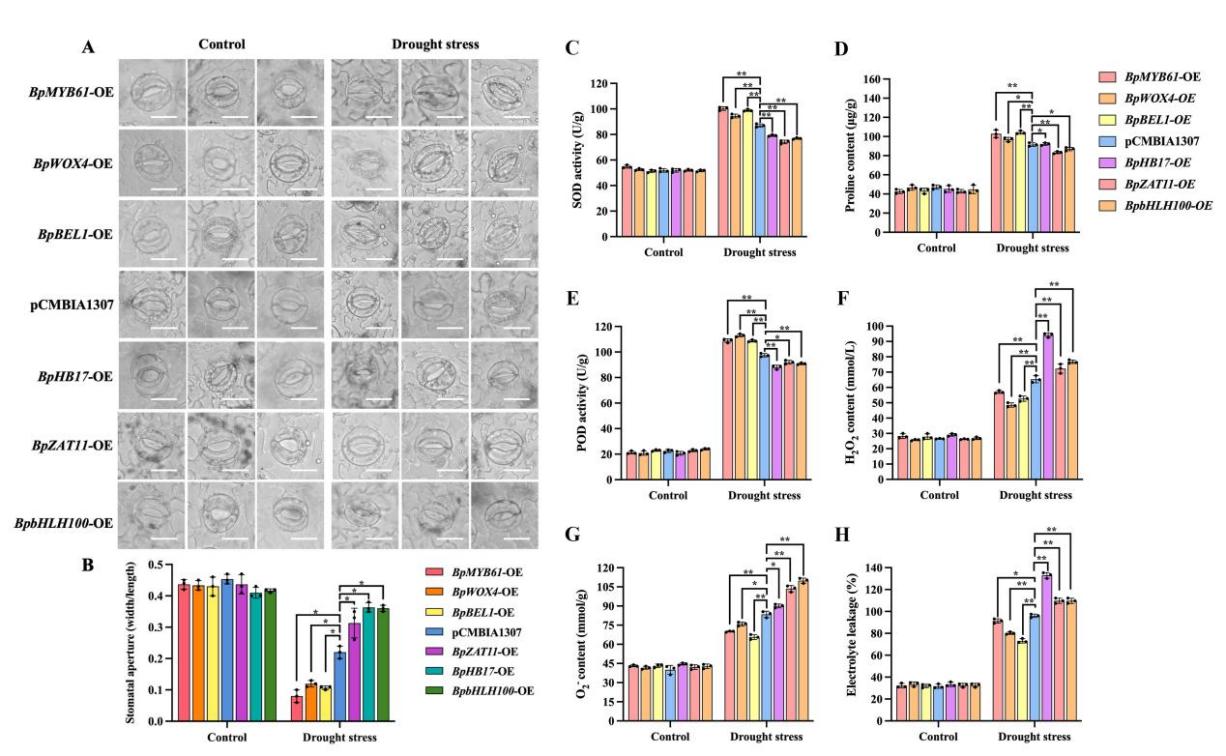


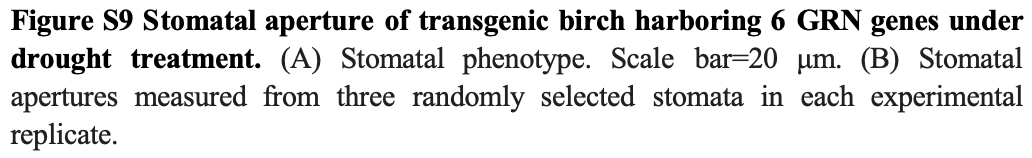


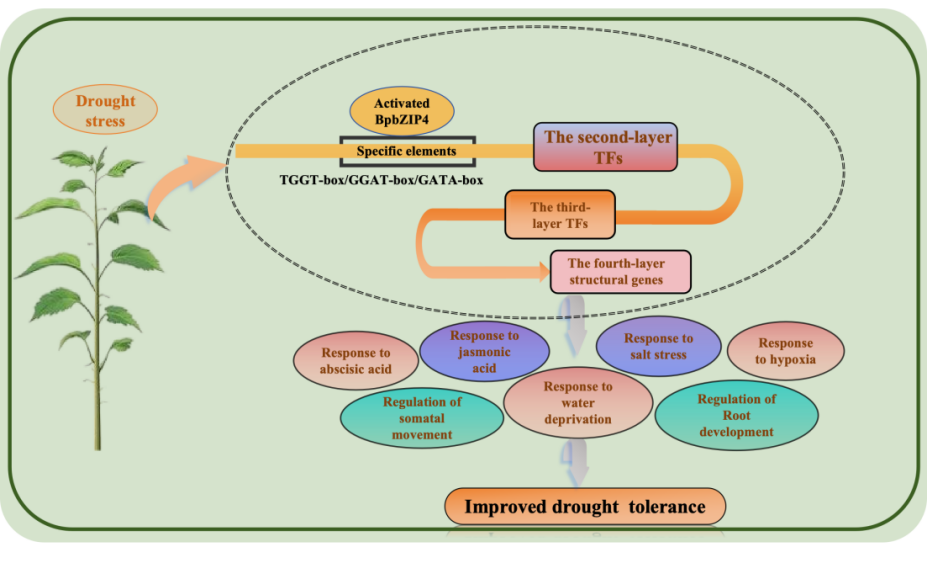


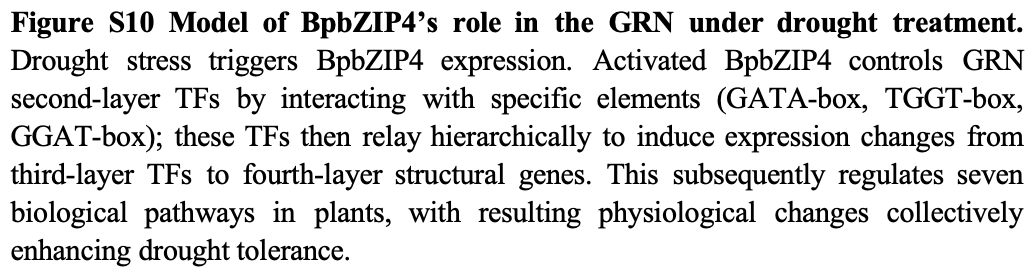


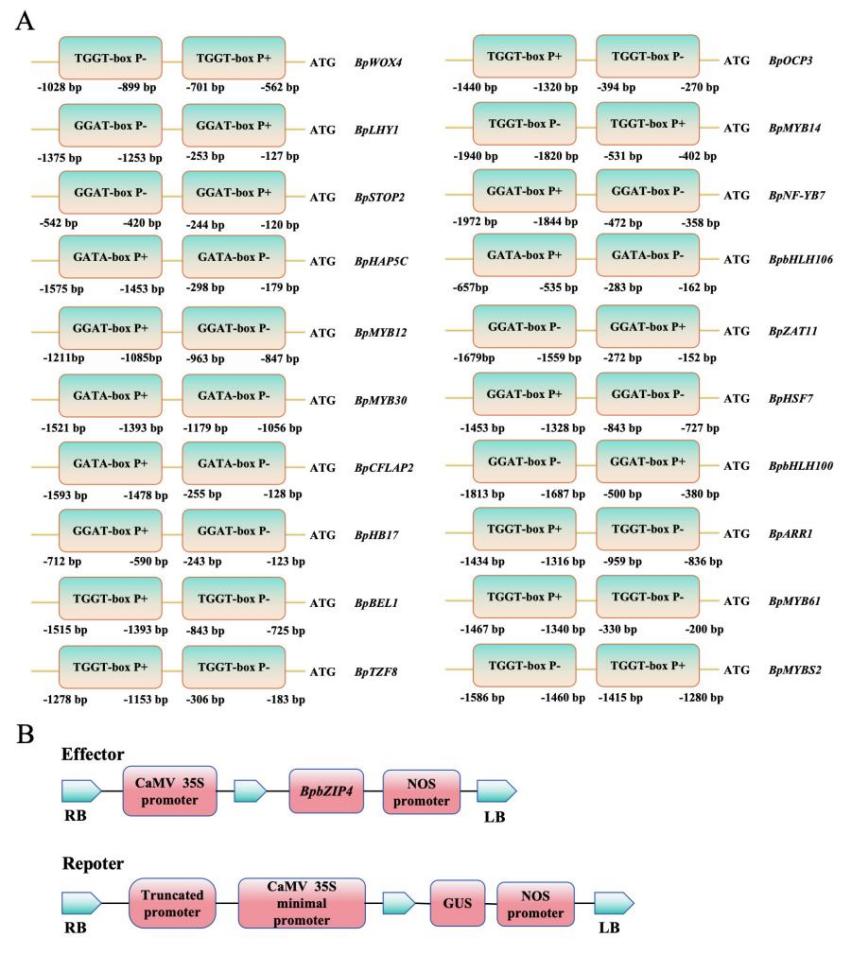


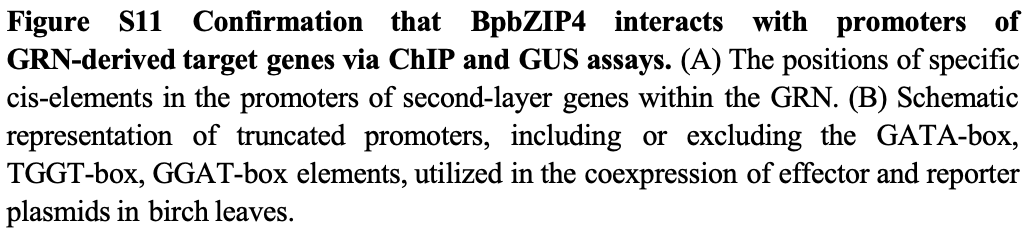

Supplement: Web_Material_uhag002 [file web_material_uhag002.zip › supplementary materials-HR-12.10.docx]
